# Stimuli‐Responsive Supramolecular Polymers of Mono‐ and Bis‐triazolylphenylazoaniline‐Functionalized Copillar[5]Arenes: With Distinctive Binding Modes

**DOI:** 10.1002/asia.202500601

**Published:** 2025-08-31

**Authors:** Chun‐Yi Yao, Yung‐Yu Chang, Reguram Arumugaperumal, Tzu‐Yi Chao, Putikam Raghunath, Ming‐Chang Lin, Wen‐Sheng Chung

**Affiliations:** ^1^ Department of Applied Chemistry National Chiao Tung University Hsinchu 30050 Taiwan; ^2^ Department of Applied Chemistry National Yang Ming Chiao Tung University Hsinchu 30050 Taiwan; ^3^ Department of Applied Chemistry, Center for Interdisciplinary Molecular Science National Yang Ming Chiao Tung University Hsinchu 30010 Taiwan

**Keywords:** Azobenzene, Click reactions, Copillar[5]arenes, Self‐assembly, Stimuli responsive, Supramolecular polymers

## Abstract

Mono‐ and bis‐triazolylphenylazoaniline‐functionalized copillar[5]arenes **1** and **2** were synthesized forming linear supramolecular polymers with dual pH and photo responsiveness. Upon acidification, copillar[5]arene **1** undergoes protonation, converting its aniline group into a primary ammonium ion (**1‐H**), which significantly enhances its supramolecular polymerization compared to **1**. Designed as an AB‐type supramolecular polymer, **1‐H** exhibits distinct polymerization behavior in contrast to neutral copillar[5]arene **2**, which forms a 1:1 supramolecular polymer with dimethoxypillar[5]arene (**DMP5**). Copillar[5]arene **2** is believed to bind **DMP5** through its cavity, linking two aniline groups from separate molecules to form a two component supramolecular polymer. The degree of polymerization of **1‐H** and the 1:1 ratio of **2** and **DMP5** can be controlled by adjusting the pH, as confirmed by pH‐ and concentration‐dependent ^1^H‐NMR and diffusion‐ordered spectroscopy (DOSY) experiments. UV–vis spectroscopy and FE‐SEM were further used to examine the effects of photoirradiation on polymer morphology and absorption at varying concentrations.

## Graphical abstract

Mono‐ and bis‐functionalized copillar[5]arenes **1** and **2** form pH‐ and photo‐responsive supramolecular polymers. Protonation of **1** enhances polymerization (**1‐H**), differing from neutral **2**, which forms a 1:1 complex with **DMP5** via cavity binding. Polymerization and morphology are tunable by pH and light, as shown by NMR, UV–vis, and FE‐SEM analyses.

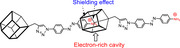

## 1 Introduction

Inspired by the remarkable self‐assembly processes in bio‐systems, significant efforts have been dedicated to designing and developing artificial supramolecular self‐assemblies with unique properties for diverse applications in chemistry, biology, and material science.^[^ ^1^ ^]^ The reversibility and stimuli‐responsiveness of self‐assembled supramolecular polymers, formed through the precise manipulation of noncovalent interactions between monomeric units have garnered considerable attention.^[^ ^2^ ^]^ These polymers can respond to various stimuli, including redox conditions, pH, temperature, solvent composition, and light irradiation.^[^ ^3^ ^]^ Relatively less effort has been directed toward incorporating photo‐responsive supramolecular polymers with controllable structural transformation, despite their potential as a power tool for constructing stimuli‐responsive supramolecular polymers with superior properties for various applications.^[^ ^4^ ^]^ The c*is‐trans* isomerization of azobenzene units can be easily triggered by UV‐vis light,^[^ ^5^ ^]^ making it not only a promising mechanism for developing photo‐switchable systems, but also a key factor in morphology changes due to its switchable configuration.^[^ ^6^ ^]^ Given their remarkable properties and multi‐functionality, azobenzene‐based system has been successfully utilized in stimuli‐responsive supramolecular polymer systems.^[^ ^7^ ^]^

Pillar[n]arenes, a new emerging class of macrocyclic hosts alongside crown ethers,^[^ ^8^ ^]^ cyclodextrins,^[^ ^9^ ^]^ calixarenes,^[^ ^10^ ^]^ resorcarenes,^[^ ^11^ ^]^ and cucurbiturils,^[^ ^12^ ^]^ were first reported in 2008 by Ogoshi and coworkers.^[^ ^13^ ^]^ Recently, the pillar[n]arenes family has played a significant role in development of supramolecular polymers through spontaneous, controllable, and well‐defined stimuli‐responsive architectures, enabling various applications such as optoelectronic devices, nanomaterials, and photonics.^[^ ^14^ ^]^ Zhao and co‐workers successfully reported stimuli‐responsive, biocompatible, pillararene‐based homogeneous supramolecular self‐assemblies for the delivery of mixed dyes in dual bioimaging in vitro.^[^ ^15^ ^]^ Yu et. al. constructed a pillar[5]arene‐based linear supramolecular polymer and a photo‐responsive supramolecular network.^[^ ^16^ ^]^ More recently, Yang and co‐workers developed a dual‐responsive pillar[5]arene‐based system featuring dynamic covalent bonding and host‐guest interactions in linear supramolecular polymers.^[^ ^17^ ^]^ Therefore, the development of photo‐switchable, stimuli‐responsive pillar[5]‐arene‐based host‐guest interactions is highly desirable in supramolecular chemistry.^[^ ^18^ ^]^ Inspired by these findings and our previous successful investigations,^[^ ^19^ ^]^ we present the design and synthesis of two novel supramolecular polymers based on the pillar[5]arene/azoaniline cation recognition motif. Furthermore, we achieved the transition between high molecular weight supramolecular polymers and relatively low molecular weight ones—i.e., control over the degree of polymerization by simply adjusting the solution pH, as confirmed by pH‐ and concentration‐dependent ^1^H NMR spectra and diffusion‐ordered spectroscopy (DOSY) experiments. The effects of photoirradiation on the morphology and absorption changes of these supramolecular polymers at different concentrations were also studied using UV‐vis spectroscopy and FE‐SEM.

## 2 Results and Discussion

**Scheme 1:**
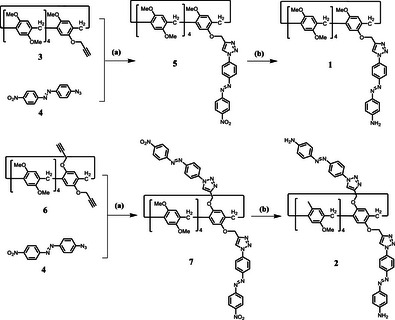
Synthesis of mono‐ and bis‐triazolylphenylazoaniline‐functionalized copillar[5]arenes **1** and **2**. Reagents and conditions: a) CuI, 1,4‐dioxane/H_2_O, reflux, 22–30 h and b) Na_2_S, 1,4‐dioxane/H_2_O, reflux, 3 h.

The syntheses of mono‐ and bis‐triazolylphenylazoaniline‐functionalized copillar[5]arenes **1** and **2**, which exhibited dual pH and photo responsive properties, are shown in Scheme 1. Copillar[5]arenes **3** ^[^ ^20^ ^]^ and **6** ^[^ ^21^ ^]^ were prepared following methods previously reported by Yang and Li. Azobenzene‐functionalized copillar[5]arenes **5** and **7** were obtained via click reactions between 4‐azido‐4′‐nitroazobenzene (**4**)^[^ ^22^ ^]^ and mono‐ and bis‐propargyl‐substituted copillar[5]arenes **3** and **6**, respectively. Subsequent Na_2_S‐mediated reduction of copillar[5]arenes **5** and **7** yielded the corresponding mono‐ and bis‐triazolylphenylazoaniline‐functionalized copillar[5]arenes **1** and **2**, with yields of 72% and 68%, respectively. The structures of compounds **1** and **2**, along with all intermediates (**3**–**7**) involved in this work, were fully characterized using ^1^H NMR, ^13^C NMR, and high‐resolution mass spectrometry (see Experimental Section and Supporting Information).

**Figure 1:**
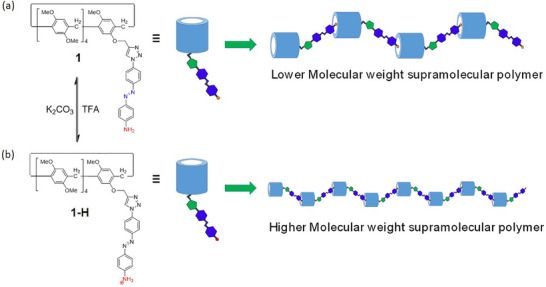
Possible supramolecular polymerization modes for a) triazolylphenylazoaniline mono‐functionalized‐pillar[5]arene **1** and b) its acidified form **1**‐**H**.

Of the two copillar[5]arenes, **1** and **2**, only mono‐triazolyl‐phenylazoaniline **1** can self‐organize into linear supramolecular polymers at high concentrations. Acidification of copillar[5]arene **1** transforms its aniline group into a primary ammonium ion, leading to **1‐H**, which significantly enhances the degree of supramolecular polymerization compared to **1** (see Figure 1b). The acidified monomer **1‐H** is designed as an AB‐type supramolecular polymer, whereas neutral copillar[5]arene **2** does not form supramolecular polymers, even at low pH or high concentrations due to intermolecular steric hindrance between the two arms of compound **2**. These findings are supported by various techniques, including concentration‐dependent ^1^H‐NMR spectroscopy, 2D‐DOSY, nuclear overhauser effect spectroscopy (NOESY), and scanning electron microscopy (SEM) experiments.

The host‐guest complexation of copillar[5]arenes **1**, **1‐H**, and **2** along with their supramolecular polymer formation was investigated using ^1^H NMR spectroscopy. Concentration‐dependent ^1^H NMR spectra of copillar[5]arene **1** (Figure S1) (600 MHz, CDCl_3_, 298 K) and its positively charged analogue **1‐H** (Figure 3) provided important insights into their self‐assembly behaviors in solution. Both complexation processes occur in fast exchange on the ^1^H NMR time scale. As the concentration of copillar[5]arene **1** in CDCl_3_ gradually increased from 2.5 to 50 mM, the signals of the triazole proton (H_i_), aryl protons (H_e_), methoxy protons (H_a_), and methylene protons (H_c_) adjacent to the triazole group all exhibited slight downfield shifts. However, the amino protons (H_b_) and azobenzene protons (H_d_) near the amino group of copillar[5]arene **1** showed significant upfield shifts due to the strong shielding effect of the pillar[5]arene cavity. These observations indicated that during the supramolecular polymerization of copillar[5]arene **1**, the pillar[5]arene cavity is fully threaded with the aniline group from an adjacent phenylazoaniline unit (Figure 1a). Similarly, complexation induced proton chemical shift changes were observed in the ^1^H NMR spectra of the acidified copillar[5]arene **1**, namely **1‐H**, at various concentrations (2.5 to 50 mM) (Figure 3). Notably, the azobenzene protons (H_d_) near the ammonium group exhibited a significant upfield shift from 6.95 to 6.45 ppm, while all methoxy protons (H_a_ and Ar‐OMe) were downfield shifted. The results suggest that the aniline/ammonium units of **1** and **1‐H**, bearing azobenzene side chains, can pass through the pillar[5]arene cavity to form linear aggregates. Due to additional electrostatic interactions between the pillar[5]arene and the ammonium group, the positively charged monomer **1‐H** forms high‐molecular‐weight supramolecular polymers more efficiently than the neutral copillar[5]arene **1** (see Figure 1a,b). Further evidence for the stronger supramolecular polymerization of **1‐H** compared to that of **1** comes from DOSY experiments (vide infra) and density functional theory (DFT) calculations (see Figures S25, S26, and Tables S2–S5).

**Figure 2:**
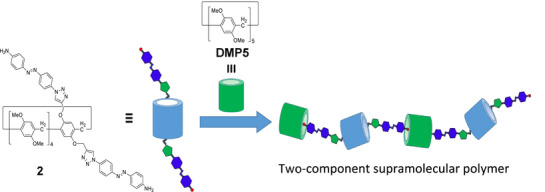
A possible two‐component supramolecular polymerization mode for di‐triazolylphenylazoaniline‐functionalized copillar[5]arene **2** with **DMP5**.

Interestingly, copillar[5]arene **2**, which has bis‐triazolyl‐phenylazoaniline groups at both ends of the pillar[5]arene, did not exhibit any signs of supramolecular polymerization as its concentration in solution increased (Figure 4). However, in the presence of a decamethoxypillar[5]arene (**DMP5**)^[^ ^13c^ ^]^ copillar[5]arene **2** efficiently formed a 1:1 supramolecular polymer thorough host–guest interactions between its phenylazoaniline groups and **DMP5**, resulting in a two‐component supramolecular polymer (see Figure 2). To investigate this interaction, NMR tubes containing host **2** (2.5 mM) and 0.2–4.0 equivalents of **DMP5** were allowed to stand at room temperature for 30 min before their ^1^H NMR spectra were recorded. Significant downfield shifts were observed in the characteristic resonances of triazole protons (H_g_) and aryl protons (H_c_) of copillar[5]arene **2**, and aryl methoxy protons (H_α_) of **DMP5** (Figure S2), confirming the formation of an interpenetrated host–guest complex between **DMP5** and host **2** (Figure 2). Additionally, the signals of the amino protons (H_a_) and the adjacent azobenzene protons (H_b_) of copillar[5]arene **2** were both upfield shifted due to the shielding effect induced by inclusion within the cavity of **DMP5**, further supporting the formation of an inclusion complex between copillar[5]arene **2** and **DMP5**.

**Figure 3:**
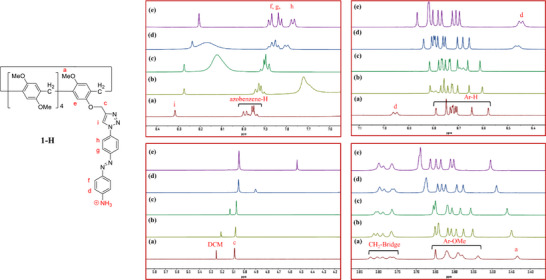
Partial ^1^H NMR spectra (600 MHz, 298 K) of pillararene **1‐H** in CDCl_3_ at various concentrations a) 2.5 mM, b) 5 mM, c) 10 mM, d) 25 mM, and e) 50 mM.

Further evidence for the formation of supramolecular polymers between copillar[5]arene **2** and **DMP5** was obtained from the ^1^H NMR spectra of a 1:1 mixture of copillar[5]arene **2** and **DMP5** at concentrations ranging from 2.5 to 50 mM in CDCl_3_ (Figure S3). As expected, increasing the concentration of the 1:1 mixture led to downfield shifts and broadening of the signals corresponding to the triazole protons (H_g_) and aryl protons (H_c_) of copillar[5]arene **2**, as well as the aryl methoxy protons (H_α_) of **DMP5**. Additionally, broadening was observed for the H_β_ proton of **DMP5**, while the H_a_ and H_b_ protons of copillar[5]arene **2** exhibited upfield shifts. These observations support the formation of a high‐molecular‐weight supramolecular polymer between copillar[5]arene **2** and **DMP5**. Similar downfield and upfield shifts along with signal broadening of pillar[5]arene proton signals have been reported by Huang and coworkers in related self‐threaded linear supramolecular polymers.^[^ ^8^, ^18^ ^]^

**Figure 4:**
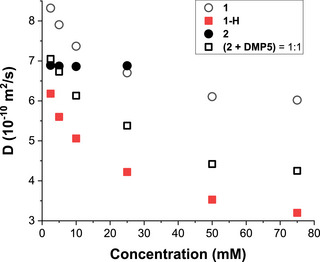
Concentration‐dependence of diffusion coefficient (*D*) (600 MHz, CDCl_3_, 298 K) of copillar[5]arenes **1** (ο), **1‐H** (■), **2** (●), and 1:1 mixture of copillar[5]arene **2** with **DMP5** (**□**).

Two‐dimensional diffusion‐ordered ^1^H NMR spectroscopy (2D‐DOSY)^[^ ^23^ ^]^ is an efficient and convenient method for evaluating the propensity of monomers to undergo self‐assembly. Therefore, concentration‐dependent DOSY experiments were conducted to investigate the self‐assembly behavior of copillar[5]arenes **1** (a neutral monomer), **1‐H** (a positively charged monomer), and **2** in forming linear supramolecular polymers in CDCl_3_ solutions (Figure 4 and Table S1). As the concentration of copillar[5]arene **1** increased from 2.5 to 75 mM, the measured weight‐average diffusion coefficient (*D*) decreased from 8.32 × 10^−10^ to 6.02 × 10^−10^ m^2^/s (28% decrease). In the cases of **1‐H**, the diffusion coefficient (*D*) decreased significantly from 6.18 × 10^−10^ to 3.20 × 10^−10^ m^2^/s (48% decrease) over the same concentration range. These results clearly indicate the gradual formation of high‐molecular‐weight, linearly extended supramolecular

Polymers from small oligomers (Figure 1b). In contrast, the diffusion coefficient (*D*) and chemical shifts in ^1^H NMR spectra of copillar[5]arene **2** remained nearly unchanged when its concentration was increased from 2.5 to 25 mM (Figure 4 and and Figure S24), suggesting that **2** does not undergo self‐assembly. This lack of polymerization is likely due to steric hindrance caused by the extruded phenylazoaniline groups at both ends of the molecule, which is supported by lowest‐energy conformations of the dimer **2** obtained from DFT calculations at the B3LYP/6–31G(d,p) level (see Figure S26b,c). Interestingly, 2D‐DOSY measurements of 1:1 mixtures of copillar[5]arene **2** and **DMP5** at various concentrations showed a steady and significant decrease in its diffusion coefficient (*D*), implying chain extension and the formation of high‐molecular‐weight polymeric structures. When the concentration of the 1:1 complex increased from 2.5 to 75 mM in CDCl_3_, the diffusion coefficient (*D*) decreased from 7.05 × 10^−10^ to 4.25 × 10^−10^ m^2^/s (40% decrease). To further investigate these self‐assembly processes, 2D Nuclear Overhauser Effect Spectroscopy (NOESY) experiments were performed on solutions of copillar[5]arenes **1** and **2** with **DMP5** at high concentrations. The results revealed correlations between the amino group of copillar[5]arene **1** and the protons on the dimethoxyhydroquinone unit of **DMP5**, confirming their interactions. Similarly, Rotating frame Overhauser Effect Spectroscopy (ROESY) experiments demonstrated NH‐π and electrostatic interactions in **1‐H**, consistent with the self‐inclusion of the positively charged ammonium ion into the cavity of pillar[5]arene (Figures S4–S8).

**Figure 5:**
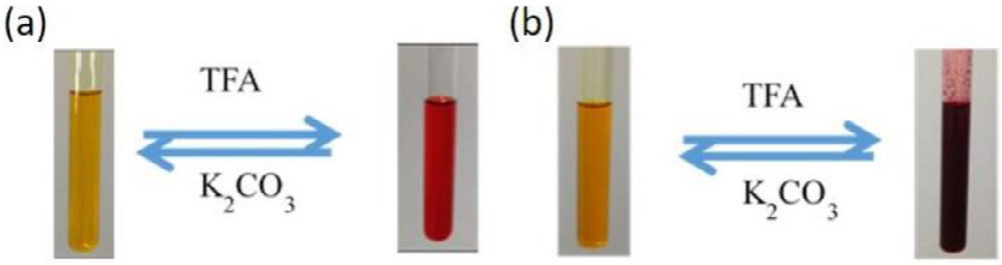
Photos of color changes of copillar[5]arenes a) **1** and b) **2** in chloroform with the addition of TFA and K_2_CO_3_.

Acid‐base controllable color changes are highly desirable and fascinating in supramolecular polymers, as externally stimuli‐responsive materials offer greater flexibility and adaptability. As expected, reversible color changes from yellow to red were observed due to the acid–base‐controlled recognition of the amino/ammonium groups in copillar[5]arenes **1** and **1‐H**. Treating copillar[5]arene **1** with acid led to protonation of its amino moiety, forming copillar[5]arene **1‐H**, which significantly enhanced supramolecular polymerization. In contrast, copillar[5]arene **2** formed a precipitate upon acid addition. However, when **DMP5**

(dimethoxypillar[5]arene) was introduced into the solution of copillar[5]arene **2**, supramolecular polymers were successfully formed. The color change from yellow to red was attributed to the ICT (Intramolecular Charge Transfer) effect and could be reversibly triggered by the addition of TFA (trifluoroacetic acid) and K_2_CO_3_ (potassium carbonate) as shown in Figure 5. To further investigate this reversible switching, we performed ^1^H NMR spectroscopy on copillar[5]arenes **1** and **2**. Upon the addition of excess amount of TFA to a solution of copillar[5]arene **2**, the resulting spectrum became highly complex due to precipitate formation in the acidic medium (Figure S10), making further analysis difficult. However, when excess TFA was added to a solution of copillar[5]arene **1**, the spectrum exhibited significant chemical shift changes, confirming the formation of **1‐H**. Specifically, the signals of triazole protons (H_i_) and methoxy protons (H_a_) in copillar[5]arene **1** shifted downfield, while the signals corresponding to the amino protons (H_b_) and the azobenzene protons (H_h_) adjacent to the amino group disappeared completely. The reversibility of this transformation was demonstrated by adding an excess amount of K_2_CO_3_ to the solution of **1‐H**, which restored the original ^1^H NMR spectrum of copillar[5]arene **1** (Figure S9). These observations confirmed the protonation of the amino group induces a color change while also enhancing the ability of copillar[5]arene **1** to form supramolecular polymers.

**Figure 6:**
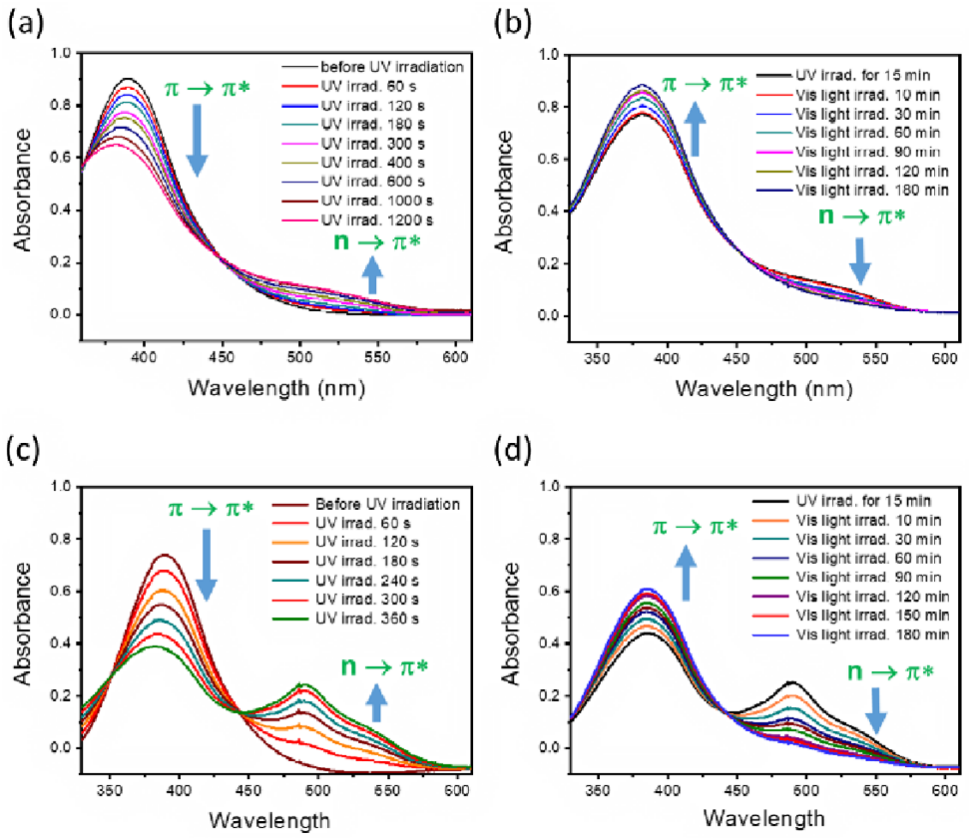
UV‐visible light absorption spectra of a) copillar[5]arene **1** (2.75 × 10^−5^ M) and c) copillar[5]arene **2** (1.5 × 10^−5^ M) irradiated with 365 nm UV light for different duration, b and d) after samples a and c) were irradiated with 365 nm UV light for 15 min and then 450 nm visible light was irradiated for different duration.

The *cis‐trans* isomerization of the azobenzene units of copillar[5]arenes **1** and **2** was further examined using UV–vis absorption spectroscopy. As shown in Figure 6, the absorption bands of monomers **1** and **2** at 390 nm decreased significantly upon irradiation with 365 nm light, accompanied by the appearance of new absorption bands between 520 and 490 nm, respectively. The absorption band at approximately 390 nm corresponds to the π → π* transition of the *trans*‐azobenzene group in copillar[5]arenes **1** and **2**, while the bands at 520 and 490 nm correspond to the n → π* transition of the *cis*‐azobenzene form in these compounds. Furthermore, when the UV‐irradiated samples of monomers **1** and **2** were exposed to visible light (450 nm), they reverted to their *trans*‐azobenzene form (see Figure 6). Notably, the *cis‐to‐trans* relaxation process required a longer irradiation time for both copillar[5]arenes **1** and **2**.

**Figure 7:**
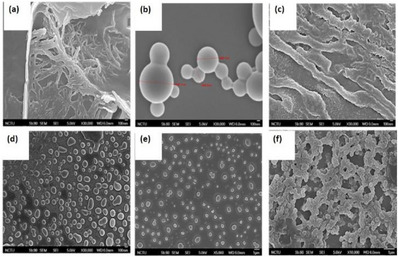
FE‐SEM images of copillar[5]arenes in CHCl_3_ a) **1** (1.0 × 10^−4^ M), b) **1‐H** (1.0 × 10^−4^ M), c) **1‐H** (1.0 × 10^−3^ M), d) **2** (5 × 10^−5^ M), e) **DMP5** (1.0 × 10^−3^ M), f) 1:1 mixture of copillar[5]arene **2** and **DMP5** (each at 5 × 10^−5^ M). Scale bar is 100 nm in (a–d) and is 1 µm in (e and f).

We were particularly interested in exploring the morphology of compounds **1**, **1‐H**, and **2**, as well as their self‐assembled supramolecular polymers using SEM. Additionally, we investigated their physical and chemical properties under UV–vis light irradiation. Copillar[5]arene **1** (100 µM in CHCl_3_) exhibited a strip‐shaped fibrous morphology (Figure 7a) with fibers that aggregated together. In contrast, the acidified form copillar[5]arene **1**‐**H**, displayed spherical aggregates with particle sizes ranging from 400 to 1000 nm at lower concentration (100 µM in CHCl_3_, Figure 7b). However, at higher concentration (1 mM in CHCl_3_) wider strip‐shaped fibers were observed (Figure 7c). On the other hand, the bis‐triazolylphenylazoaniline modified copillar[5]arene **2** (50 µM in CHCl_3_) exhibited a spherical and block‐like morphology (Figure 7d). Similarly, **DMP5** (1 mM in CHCl_3_) also displayed an independent block‐like distribution (Figure 7e). Neither copillar[5]arene **2** nor **DMP5** exhibited an obvious cross‐linked morphology. However, SEM analysis of a 1:1 mixture of copillar[5]arene **2** with **DMP5** in chloroform (50 µM in CHCl_3_) revealed a distinct cross‐linked block morphology (Figure 7f), which was markedly different from the individual morphologies of copillar[5]arene **2** and **DMP5**. The observed morphologies in the SEM images can be explained by the commonly accepted idea that highly directional intermolecular interactions—such as hydrogen bonding, cationic‐π, CH–π, or π–π interactions—favored the formation of organized nanobelt or nanofiber structures. The SEM results suggest that copillar[5]arene **2** interacts with **DMP5** to form supramolecular polymers, consistent with findings from concentration‐dependent ^1^H NMR and DOSY experiments (vide supra).

DOSY experiments (600 MHz, CDCl_3_, 298 K) were conducted to determine whether the degree of supramolecular polymerization of acidified copillar[5]arene **1‐H** is affected by the photo‐induced cis‐trans isomerization of its azobenzene group. The diffusion coefficient (*D*) of acidified copillar[5]arene **1‐H** (50 mM in CDCl_3_) was measured as 3.71 × 10^−10^ m^2^/s. After irradiating the same solution with a Rayonet photoreactor (350 nm, 128 W) for 1 h, the diffusion coefficient remained unchanged at 3.71 × 10^−10^ m^2^/s. As evident from the DOSY measurements, the diffusion coefficient (*D*) of copillar[5]arene **1‐H** before and after ultraviolet irradiation remained the same, indicating that the *cis‐trans* isomerization of azobenzene does not influence the self‐assembly ability of acidified copillar[5]arene **1‐H** to form supramolecular polymers. Additionally, SEM images were taken to examine whether the morphology of copillar[5]arene **1‐H** changed under ultraviolet light excitation. The SEM images of the supramolecular polymers of **1‐H** exhibited a spherical connected structure (Figure S11a) and no significant morphological changes were observed after UV‐light irradiated for 1 h (Figure S11b). These results demonstrated that the cis‐trans isomerization of the azobenzene in acidified copillar[5]arene **1‐H** does not affect the degree of supramolecular polymerization, nor does it significantly alter its morphology. Although the azobenzene substituent is attached at the end of the pillararenes, the degree of polymerization and the morphology of supramolecular polymers of **1‐H** remained largely unchanged despite the transformation of the azobenzene group from *trans‐to‐cis*.

We then investigated whether the two‐component supramolecular polymer formed by copillar[5]arene **2** and **DMP5** undergoes changes in the degree of polymerization due to the cis‐trans isomerization of the azobenzene group in copillar[5]arene **2**. First, a DOSY experiment (600 MHz, CDCl_3_, 298 K) was conducted to measure any changes in the diffusion coefficient before and after UV‐light irradiation. The diffusion coefficient of a 1:1 solution of copillar[5]arene **2** and **DMP5** (each at 50 mM in CDCl_3_) was determined to be 4.348 × 10^−10^ m^2^/s. After irradiating the same sample with 350 nm light for 1 hour, the diffusion coefficient remained nearly unchanged at 4.351 × 10^−10^ m^2^/s. As evident from the DOSY experiments, the diffusion coefficient of the 1:1 solution of copillar[5]arene **2** and **DMP5** before and after UV irradiation showed no significant change (within experimental error), indicating that the *cis‐trans* isomerization of the azobenzene groups in copillar[5]arene **2** does not affect their ability to self‐assemble into supramolecular polymers.

**Figure 8:**
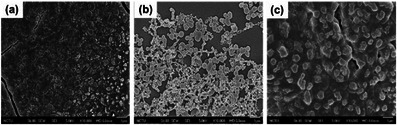
FE‐SEM images of 1:1 mixture of copillar[5]arene **2** and **DMP5** (each at 100 µM in chloroform): a) in free state, b) after UV 365 nm irradiation for 1 h, and c) after visible light irradiation of sample (b) for 20 h; scale bar is 1 µm for all figures.

SEM was then used to examine whether the morphology of the two component supramolecular polymers formed by a 1:1 mixture of copillar[5]arene **2** and **DMP5** (each at 100 µM in chloroform) changes under ultraviolet light irradiation. The SEM images revealed that before irradiation, the 1:1 mixture of copillar[5]arene **2** and **DMP5** exhibited a block‐like morphology (Figure 8a). However, after irradiation with ultraviolet light for 1 h, the morphology transformed into spherical aggregates (Figure 8b). When the mixture was subsequently irradiated with visible light for 24 h, its morphology reverted to the original block‐like structure (Figure 8c). Although, the degree of polymerization of the two component supramolecular polymers remained unchanged after ultraviolet light irradiation, notable morphological changes were observed. It is speculated that the bilaterally substituted azoanilines in copillar[5]arene **2** form end‐to‐end supramolecular polymers with **DMP5** (see Figure 2). Upon ultraviolet irradiation, the *trans‐to‐cis* isomerization of the azoanilines groups induces significant structural changes. This geometric alteration in the bis‐azoanilines within the 1:1 mixture of copillar[5]arene **2** and **DMP5** affects intermolecular interactions, leading to more pronounced morphological changes compared to copillar[5]arene **1**, which contains only single‐sided azoaniline substitution. While the geometry of copillar[5]arene **2** changes upon irradiation, its ability to associate with **DMP5** remains unaffected, resulting in no significant change in the degree of polymerization.

## 3 Conclusion

Based on NMR spectra at different concentrations, along with NOESY and ROESY experiments, we speculate that copillar[5]arene **1** forms supramolecular polymers through self‐complexation of its amine group within its own cavity, leading to a linear assembly. Similarly, acidified copillar[5]arene **1**‐**H** follows a comparable mechanism, where the ammonium ion is complexed within its own cavity and assembles into supramolecular polymers with higher molecular weights. DFT calculations on the binding energy of dimer **1**‐**H** and dimer **2** is calculated to be −22.1 and −6.5 kcal/mol, respectively, confirming the argument above (Figures S25, S26 and Tables S2‐S5). The binding mode of copillar[5]arene **2** and **DMP5** is speculated to involve the cavity of **DMP5** connecting the amine groups of two copillar[5]arene **2** molecules, forming a linear, two component supramolecular polymer. Furthermore, UV‐vis spectroscopy confirmed that the azobenzene groups in copillar[5]arenes **1** and **2** undergo *cis‐trans* isomerization upon UV‐vis light irradiation. However, the degree of polymerization of the supramolecular polymers, once formed is not influenced by the azobenzene configuration. SEM analysis was conducted to examine the microscopic morphology after irradiation. The morphology of acidified copillar[5]arene **1**‐**H** remained largely unchanged after ultraviolet light irradiation, whereas the 1:1 mixture of copillar[5]arene **2** and **DMP5** transformed into a spherical aggregate upon ultraviolet light irradiation. This morphological change is speculated to result from the structural transition of azobenzene from trans to cis, which alters intermolecular interactions within the system. Upon exposure to visible light, the morphology reverted to its original block‐like aggregation.

## 4 Experimental Section

### 4.1 Materials and Methods

All reagents were commercially available and used as supplied without further purification. ^1^H NMR and DEPT spectra were measured with 400 and 600 MHz spectrophotometers with the residual solvent peaks (usually CHCl_3_, DMSO), or TMS as the internal standard. Natural abundance ^13^C NMR spectra were recorded using pulse Fourier transform techniques with 400 and 600 MHz spectrophotometers operating at 100 and 150 MHz, respectively. Coupling constants (*J*) were reported in hertz (Hz). Detailed NMR assignments of compounds were done with the aid of ^1^H‐ and ^13^C‐NMR, 2D‐NOESY, 2D‐ROESY, and DOSY experiments. High‐resolution mass spectrometry (HRMS) was obtained with a magnetic sector type analyzer using ESI, EI, and FAB methods. UV‐vis spectra were recorded with an Agilent 8453 spectrophotometer and solvents were of HPLC grade. Illumination experiments were performed in a Rayonet RPR‐100 photochemical reactor. Melting points were determined using a Yanaco MP‐500D apparatus without correction.

### 4.2 Synthesis Procedure

The following known compounds were synthesized according to the reported procedures: **3**,^[^ ^20^ ^]^ **4**,^[^ ^22^ ^]^ **6**,^[^ ^21^ ^]^ and **DMP5**.^[^ ^13^ ^]^

**Synthesis of 1‐methoxy‐4‐((1‐(4‐((E)‐(4‐nitro‐phenyl)‐diazenyl)‐phenyl)‐1*H*‐1,2,3‐triazol‐4‐yl)‐methoxy)‐methoxycopillar[5]arene, 5**.

A mixture of compounds **3** ^[^ ^20^ ^]^ (0.26 g, 0.34 mmol), **4** ^[^ ^22^ ^]^ (0.18 g, 0.67 mmol), and CuI (0.11 g, 0.58 mmol) in mixed solvent of 1,4‐dioxane (45 mL) and water (3 mL) was stirred under reflux for 22 h. After evaporation of the solvent, the mixture was washed with water (3 × 50 mL) and extracted with chloroform (3 × 50 mL). The organic phase was dried over MgSO_4_ and the solvent was removed under reduced pressure. The residue was purified by silica gel column chromatography with a gradient polarity (ethyl acetate/*n*‐hexane = 1/40 to 1/5) as eluent to give a dark orange powder **5** (0.22 g, 63%). mp: 143–145 °C; *R*_f_ = 0.1 (ethyl acetate/ *n*‐hexane = 1/1); ^1^H NMR (400 MHz, CDCl_3_, ppm): *δ*_H_ 8.41 (d, *J* = 8.6 Hz, 2H), 8.17 (d, *J* = 8.7 Hz, 2H), 8.15 (s, 1H), 8.09 (d, *J* = 8.6 Hz, 2H), 8.01 (d, *J* = 8.7 Hz, 2H), 6.91 (s, 1H), 6.81–6.70 (m, 8H), 6.68 (s, 1H), 5.10 (s, 2H), 3.84–3.75 (m, 10H), 3.69–3.57 (m, 24H), 3.45 (s, 3H); ^13^C NMR (100 MHz, CDCl_3_, ppm): *δ*_C_ 155.3 (Cq), 151.8 (Cq), 151.4 (Cq), 150.9 (Cq), 150.9 (Cq), 150.8 (Cq), 149.4 (Cq), 148.9 (Cq), 146.4 (Cq), 139.3 (Cq), 128.6 (Cq), 128.5 (Cq), 128.4 (Cq), 128.2 (Cq), 128.2 (Cq), 128.0 (Cq), 124.8 (CH), 123.6 (CH), 120.8 (CH), 120.5 (CH), 115.2 (CH), 114.3 (CH), 114.2 (CH), 114.1 (CH), 114.1 (CH), 114.0 (CH), 114.0 (CH), 62.8 (CH_2_), 55.9 (CH_3_), 55.9 (CH_3_), 55.8 (CH_3_), 55.8 (CH_3_), 55.7 (CH_3_), 55.7 (CH_3_), 53.1 (CH_2_), 30.0 (CH_2_), 29.7 (CH_2_), 29.6 (CH_2_), 29.5 (CH_2_); HRMS (ESI) *m/z* calcd for C_59_H_59_N_6_O_12_ 1043.4185; found 1043.4211.

**Synthesis of 1‐methoxy‐4‐((1‐(4‐((E)‐(4‐aminophenyl)‐diazenyl)‐phenyl)‐1*H*‐1,2,3‐triazol‐4‐yl)methoxy)methoxy‐copillar[5]arene, 1**.

A mixture of pillararene **5** (0.2 g, 0.19 mmol) and Na_2_S (0.16 g, 2.05 mmol) in mixed solvent of 1,4‐dioxane (30 mL) and water (50 mL) was stirred under reflux for 3 h. After evaporation of the solvent, the mixture was washed with water (3 × 50 mL) and extracted with dichloromethane (3 × 50 mL). The organic phase was dried over MgSO_4_ and the solvent was removed under reduced pressure. The residue was purified by silica gel column chromatography using gradient polarity (ethyl acetate/*n*‐hexane = 1/5 to 1/1) as eluent to give a yellow solid **1** (0.14 g, 72%). mp: 126–128 °C; *R*_f_ = 0.38 (ethyl acetate/*n*‐hexane = 1/1); ^1^H NMR (400 MHz, CDCl_3_, ppm): *δ*_H_ 8.10 (s, 1H), 8.02 (d, *J* = 8.7 Hz, 2H), 7.89 (d, *J* = 8.7 Hz, 2H), 7.85 (d, *J* = 8.7 Hz, 2H), 6.91 (s, 1H), 6.80 (s, 1H), 6.78 (s, 1H), 6.78–6.75 (m, 5H), 6.74 (s, 1H), 6.72 (s, 1H), 6.71 (s, 1H), 6.67 (s, 1H), 5.09 (s, 2H), 4.13 (s, 2H), 3.82 (s, 2H), 3.80 (s, 2H), 3.78 (s, 2H), 3.78 (s, 2H), 3.76 (s, 2H), 3.68 (s, 3H), 3.66 (s, 3H), 3.65 (s, 3H), 3.64 (s, 3H), 3.63 (s, 3H), 3.62 (s, 3H), 3.60 (s, 3H), 3.58 (s, 3H), 3.43 (s, 3H); ^13^C NMR (100 MHz, CDCl_3_, ppm): *δ*_C_ 152.7 (Cq), 151.1 (Cq), 150.9 (Cq), 150.9 (Cq), 150.9 (Cq), 150.8 (Cq), 150.8 (Cq), 150.8 (Cq), 150.2 (Cq), 149.5 (Cq), 146.0(Cq), 145.4(Cq), 137.3 (Cq), 128.6 (Cq), 128.5 (Cq), 128.5 (Cq), 128.3 (Cq), 128.3 (Cq), 128.2 (Cq), 128.1 (Cq), 128.1 (Cq), 128.0 (Cq), 125.5 (CH), 123.7 (CH), 120.8 (CH), 120.6 (CH), 115.2 (CH), 114.6 (CH), 114.4 (CH), 114.3 (CH), 114.3 (CH), 114.2 (CH), 114.1 (CH), 114.1 (CH), 62.9 (CH_2_), 56.0 (CH_3_), 55.9 (CH_3_), 55.9 (CH_3_), 55.8 (CH_3_), 55.8 (CH_3_), 55.7 (CH_3_), 55.7 (CH_3_), 30.0 (CH_2_), 29.7 (CH_2_), 29.7 (CH_2_), 29.6 (CH_2_), 29.6 (CH_2_); HRMS (ESI) *m/z* calcd for C_59_H_61_N_6_O_10_ 1013.4444; found 1013.4405.

**Synthesis of 1,4‐bis((1‐(4‐((E)‐(4‐nitrophenyl)diazenyl) phenyl)‐1*H*‐1,2,3‐triazol‐4‐yl)‐methoxy)methoxycopillar‐[5]arene, 7**.

A mixture of compounds **6** ^[^ ^21^ ^]^ (0.3 g, 0.37 mmol), **4** (0.26 mg, 0.97 mmol) and CuI (0.2 g, 1.05 mmol) in mixed solvent of THF (45 mL) and water (3 mL) was stirred under reflux for 30 h. After evaporation of the solvent, the mixture was washed with water (3 × 50 mL) and extracted with chloroform (3 × 50 mL). The organic phase was dried over MgSO_4_ and the solvent was removed under reduced pressure. The residue was purified by recrystallization with dichloromethane and methanol to give an orange red powder **7** (0.26 g, 53%). mp: 167–168 °C; *R*_f_ **= 0.43 (dichloromethane); ^1^H NMR (400 MHz, CDCl_3_, ppm): *δ*_H_ 8.42 (d, *J* = 8.8 Hz, 4H), 8.18 (d, *J* = 8.6 Hz, 4H), 8.17 (s, 2H), 8.09 (d, *J* = 8.8 Hz, 4H), 8.01 (d, *J* = 8.6 Hz, 4H), 6.96 (s, 2H), 6.73 (s, 2H), 6.72 (s, 2H), 6.72 (s, 2H), 6.70 (s, 2H), 5.19 (d, *J* = 12.2 Hz, 2H), 5.08 (d, *J* = 12.2 Hz, 2H), 3.97 (s, 1H), 3.93 (s, 1H), 3.80–3.73 (m, 8H), 3.61–3.58 (m, 18H), 3.47 (s, 6H); ^13^C NMR (100 MHz, CDCl_3_, ppm): *δ*_C_ 155.3 (Cq), 151.8 (Cq), 150.9 (Cq), 150.9 (Cq), 150.8 (Cq), 150.0 (Cq), 149.0 (Cq), 146.2 (Cq), 139.3 (Cq), 128.9 (Cq), 128.7 (Cq), 128.3 (Cq), 128.1 (Cq), 127.8 (Cq), 125.0 (CH), 124.8 (CH), 123.6 (CH), 120.8 (CH), 120.5 (CH), 115.4 (CH), 114.4 (CH), 114.2 (CH), 114.1 (CH), 62.8 (CH_2_), 56.0 (CH_3_), 56.0 (CH_3_), 55.9 (CH_3_), 55.8 (CH_3_), 30.0 (CH_2_), 29.7 (CH_2_). HRMS (ESI) *m/z* calcd for C_73_H_67_N_12_O_14_ 1335.4894; found 1335.4917.

**Synthesis of 1,4‐di((1‐(4‐((E)‐(4‐aminophenyl)diazenyl) phenyl)‐1*H*‐1,2,3‐triazol‐4‐yl)ethoxy)methoxycopillar‐[5]arene, 2**.

A mixture of pillararene **7** (0.3 g, 0.23 mmol) and Na_2_S (0.36 g, 4.61 mmol) in mixed solvent of 1,4‐dioxane (50 mL) and water (10 mL) was stirred under reflux for 3 h. After evaporation of the solvent, the mixture was washed with water (3 × 50 mL) and extracted with dichloromethane (3 × 50 mL). The organic phase was dried over MgSO_4_ and the solvent was removed under reduced pressure. The residue was purified by silica gel column chromatography using gradient polarity (ethyl acetate/*n*‐hexane = 1/10 to 1/1) as eluent to give a yellow solid **2** (0.2 g, 68%). mp: 152–154 °C; *R*_f_ **= 0.75 (ethyl acetate/*n*‐hexane = 3/1); ^1^H NMR (400 MHz, CDCl_3_, ppm): *δ*_H_ 8.13 (s, 2H), 8.02 (d, *J* = 8.7 Hz, 4H), 7.90 (d, *J* = 8.8 Hz, 4H), 7.85 (d, *J* = 8.7 Hz, 4H), 6.96 (s, 2H), 6.78–6.74 (m, 6H), 6.73 (s, 4H), 6.70 (s, 2H), 5.18 (d, *J* = 12.2 Hz, 2H), 5.07 (d, *J* = 12.2 Hz, 2H), 4.12 (s, 4H), 3.96 (s, 1H), 3.93 (s, 1H), 3.82–3.71 (m, 8H), 3.61 (s, 12H), 3.59 (s, 6H), 3.46 (s, 6H); ^13^C NMR (100 MHz, CDCl_3_, ppm): *δ*_C_ 152.7 (Cq), 151.0 (Cq), 150.9 (Cq), 150.9 (Cq), 150.8 (Cq), 150.2 (Cq), 150.0 (Cq), 145.9 (Cq), 145.4 (Cq), 137.3 (Cq), 128.9 (Cq), 128.6 (Cq), 128.3 (Cq), 128.1 (Cq), 127.9 (Cq), 125.5 (CH), 123.8 (CH), 120.8 (CH), 120.6 (CH), 115.4 (CH), 114.6 (CH), 114.4 (CH), 114.4 (CH), 114.1 (CH), 114.1 (CH), 62.8 (CH_2_), 56.0 (CH_3_), 56.0 (CH_3_), 55.8 (CH_3_), 55.8 (CH_3_), 30.0 (CH_2_), 29.7 (CH_2_); HRMS (ESI) *m/z* calcd for C_73_H_71_N_12_O_10_ 1275.5411; found 1275.5431.

## Conflict of Interests

The authors declare no conflict of interests.

## Supporting information



Supporting Information

## Data Availability

The data underlying this study are available in the published article and its Supporting Information. Supporting information for this article is available on the *WWW* under https://doi.org/10.1003/asia.2025xxyyz.
