# Supplementary material for: Stimuli‐Responsive Supramolecular Polymers of Mono‐ and Bis‐triazolylphenylazoaniline‐Functionalized Copillar[5]Arenes: With Distinctive Binding Modes
Source: Chem Asian J. 2025 Aug 31;20(21):e00601. doi: 10.1002/asia.202500601 (PMC12584021; doi:10.1002/asia.202500601)
Supplement: Supplementary file 1 — Supporting Information [file ASIA-20-e00601-s001.pdf]

Supporting Information

Stimuli Responsive Supramolecular Polymers of Mono- and Bis-triazolyl-phenylazoanilines–Functionalized Copillar[5]arenes: With Distinctive Binding Modes

Chun-Yi Yao,[a] Yung-Yu Chang,a Reguram Arumugaperumal,[a] Tzu-Yi Chao,[b] Putikam Raghunath,[c] Ming-Chang Lin,[c] and Wen-Sheng Chung*[, a, b]

[a] *Department of Applied Chemistry, National Chiao Tung University, Hsinchu, Taiwan 30010, Republic of China*

[b] *Department of Applied Chemistry, National Yang Ming Chiao Tung University, Hsinchu, Taiwan 30010, Republic of China;* wschung@nycu.edu.tw

[c] *Center for Interdisciplinary Molecular Science, Department of Applied Chemistry, National Yang Ming Chiao Tung University, Hsinchu, Taiwan 30050*

Table of Contents

**Content**	**Page**
Synthesis overview of compounds **1** and **2**.	S2
**Figures S1-S24:** ^{1}H- and ^{13}C-NMR spectra, DOSY, NOESY, ROESY and FE-SEM of Compounds **1**, **1-H**, **2**, concentration-dependent **2**, and 1:1 ratio of **2** and **SMP5**.	S3-S16
**Table S1**: Concentration dependence of the diffusion coefficient *D* of **1**, **1-H**, **2**, and 1:1 ratio of **2** and **SMP5**.	S17
**Figure S25**: Geometry optimizations of (a) 1-H, (b) side view of $(1\text{-H})_2$, and (c) top view of $(1\text{-H})_2$, computed using Gaussian 16 under B3LYP/6-31G(d,p) level in the gas phase.<br>**Figure S26**: Geometry optimizations of (a) 2, (b) side view of dimer 2, and (c) top view of dimer 2, computed using Gaussian 16 under B3LYP/6-31G(d,p) level in the gas phase.	S18–S19
Theoretical study and Optimized Cartesian structures at the B3LYP/6-31G (d,p) level. **Table S2**: monomer **1-H**; **Table S3**: Dimer **(1-H)$_2$** (Charge = +2); **Table S4**: monomer **1** (neutral); and **Table S5**: Dimer **1** (neutral).	S20–S31

Synthesis overview

The following compounds were synthesized according to the reported procedures: **3**[20], **4**[22], **6**[21], and **DMP5**.[13]

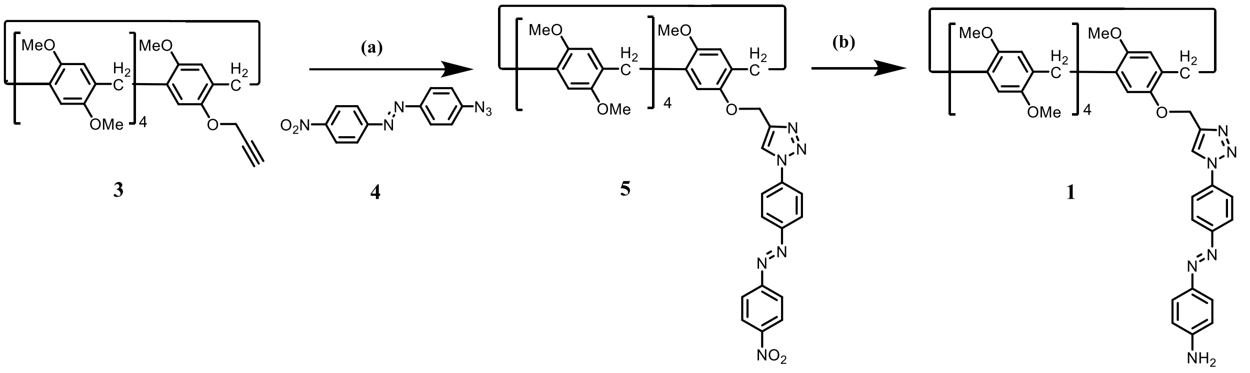

Scheme S1. Synthesis of pillararene **1**; Reagents and conditions: (a) CuI, 1,4-dioxane/H_2O, reflux 22 h, 63%; (b) Na_2S, 1,4-dioxane/H_2O, reflux 3 h, 72 %.

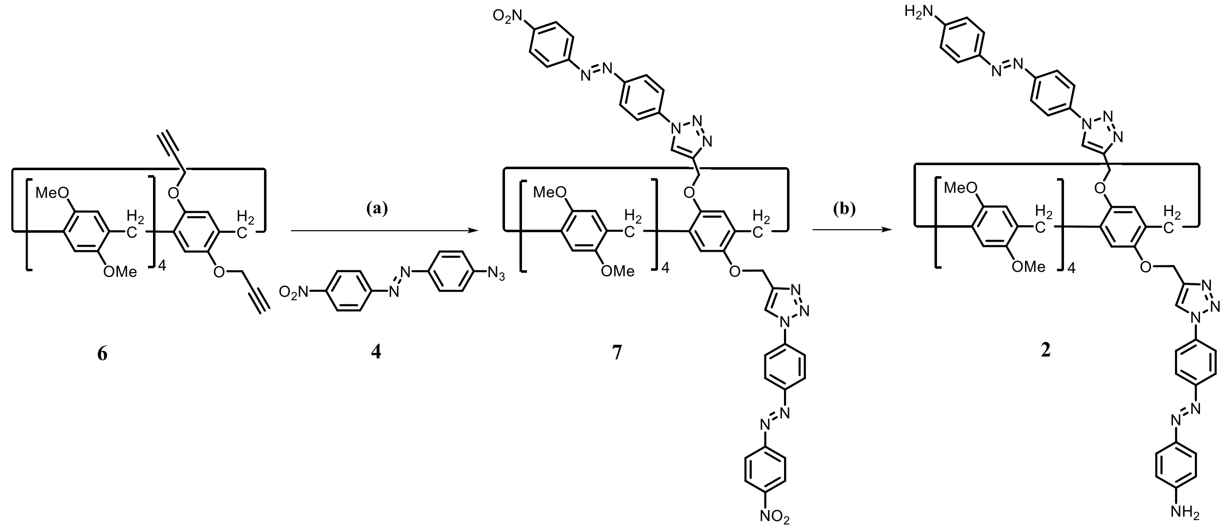

Scheme S2. Synthesis of pillararene **2**; Reagents and conditions: (a) CuI, THF/H_2O, reflux 30 h, 53%; (b) Na_2S, 1,4-dioxane/H_2O, reflux 3 h, 68%.

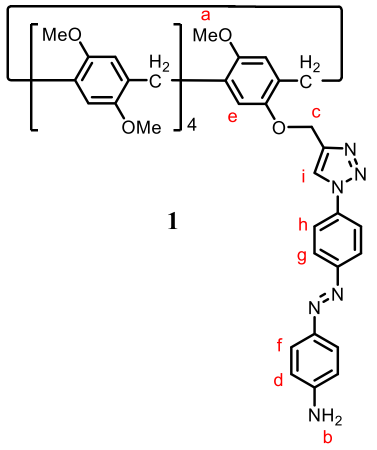

1

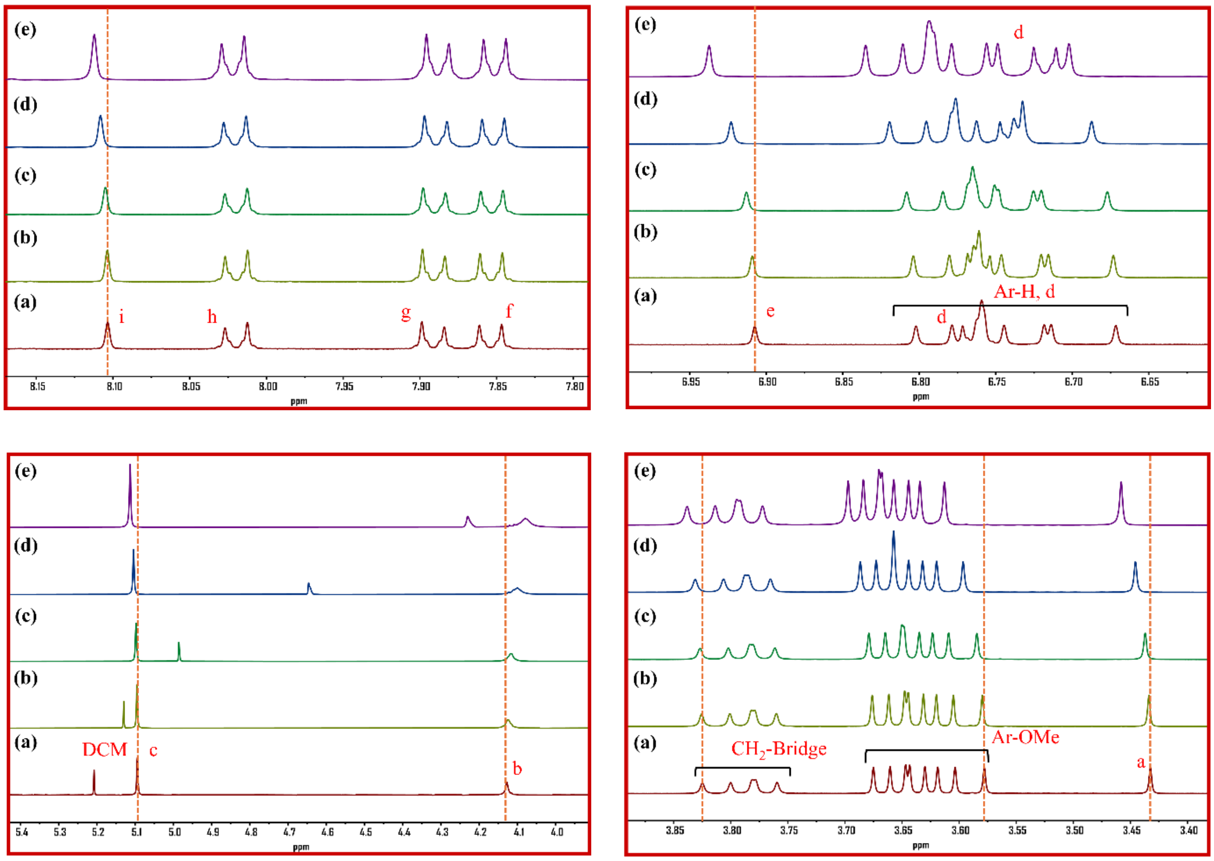

Figure S1. Partial ^{1}H **NMR** spectra (600 MHz, 298 K) of pillararene **1** in $CDCl_3$ at various concentrations (a) 2.5 mM, (b) 5 mM, (c) 10 mM, (d) 25 mM, and (e) 50 mM.

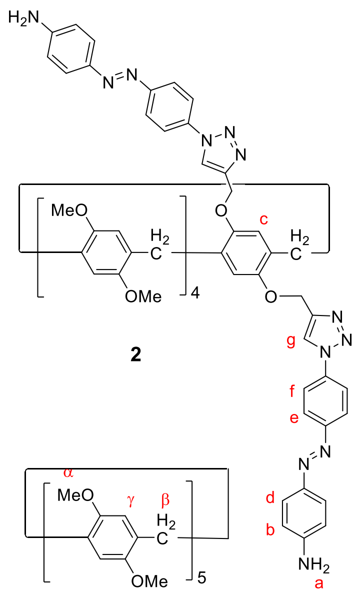

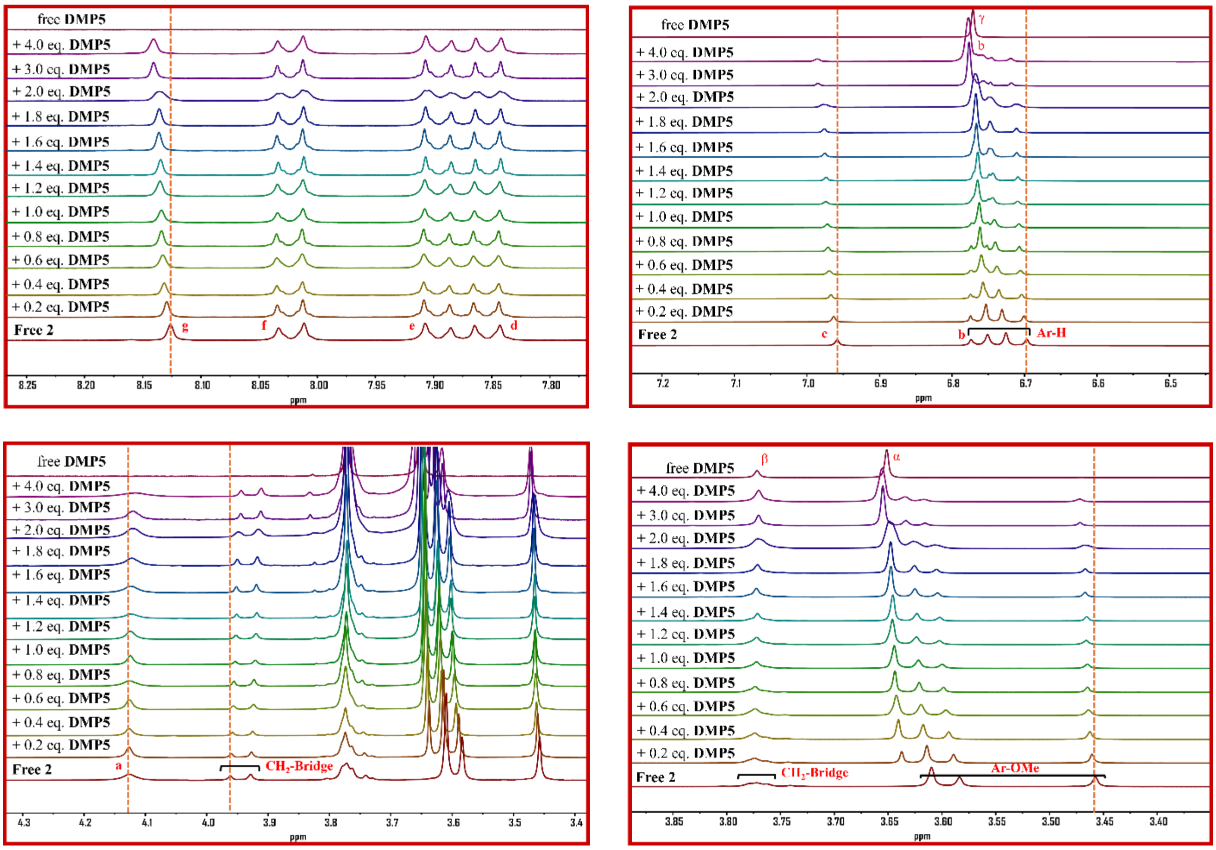

Figure S2. Partial ^{1}H NMR spectra (400 MHz, 298 K) of pillararene **2** in $CDCl_3$ (2.5 mM) with the addition of various equiv (0.2–4.0) of **DMP5**.

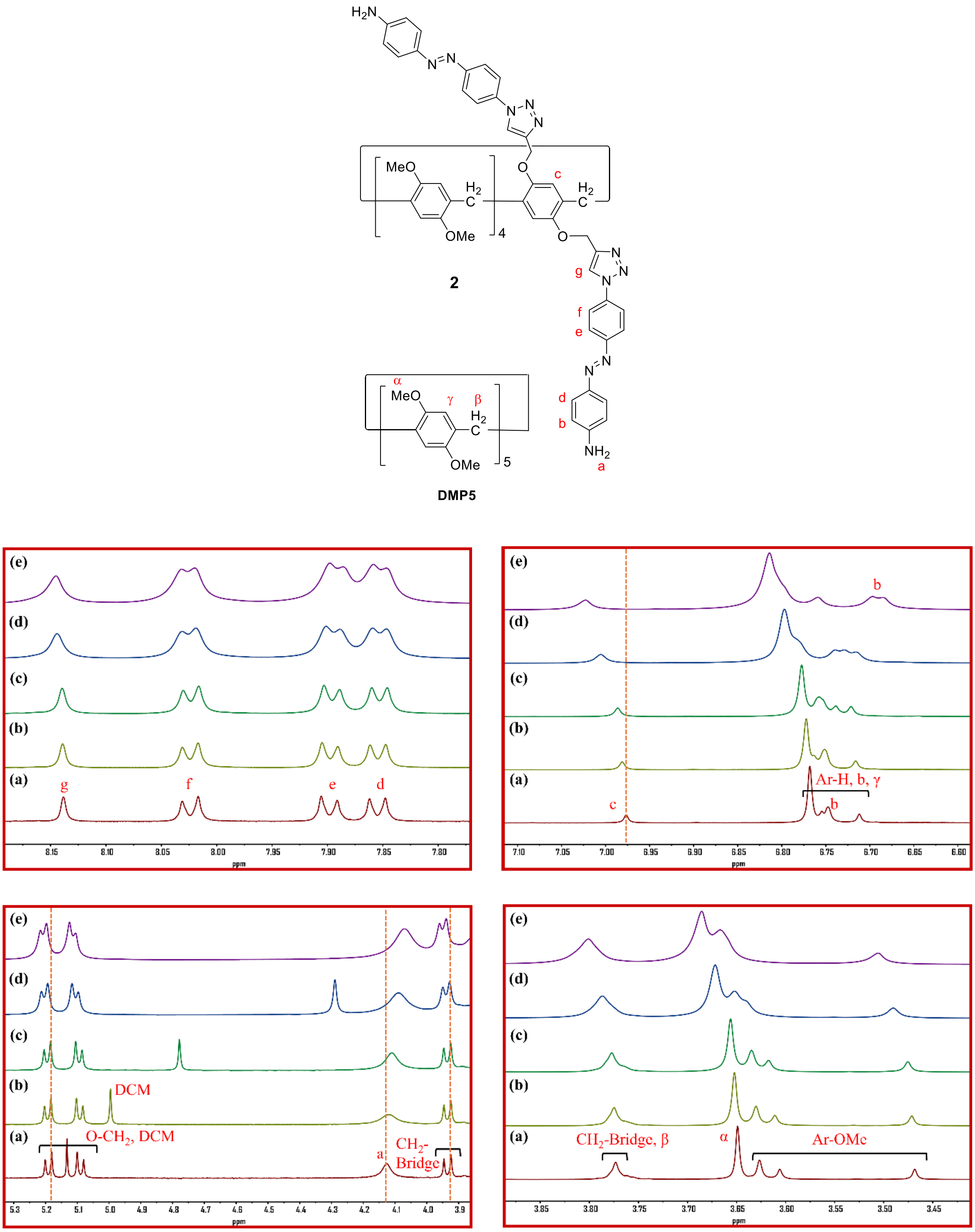

Figure S3. Partial ^{1}H **NMR** spectra (600 MHz, 298 K) of 1:1 molar mixture of pillararenes **2** and **DMP5** in $CDCl_3$ at various concentrations: (a) 2.5 mM, (b) 5 mM, (c) 10 mM, (d) 25 mM, and (e) 50 mM.

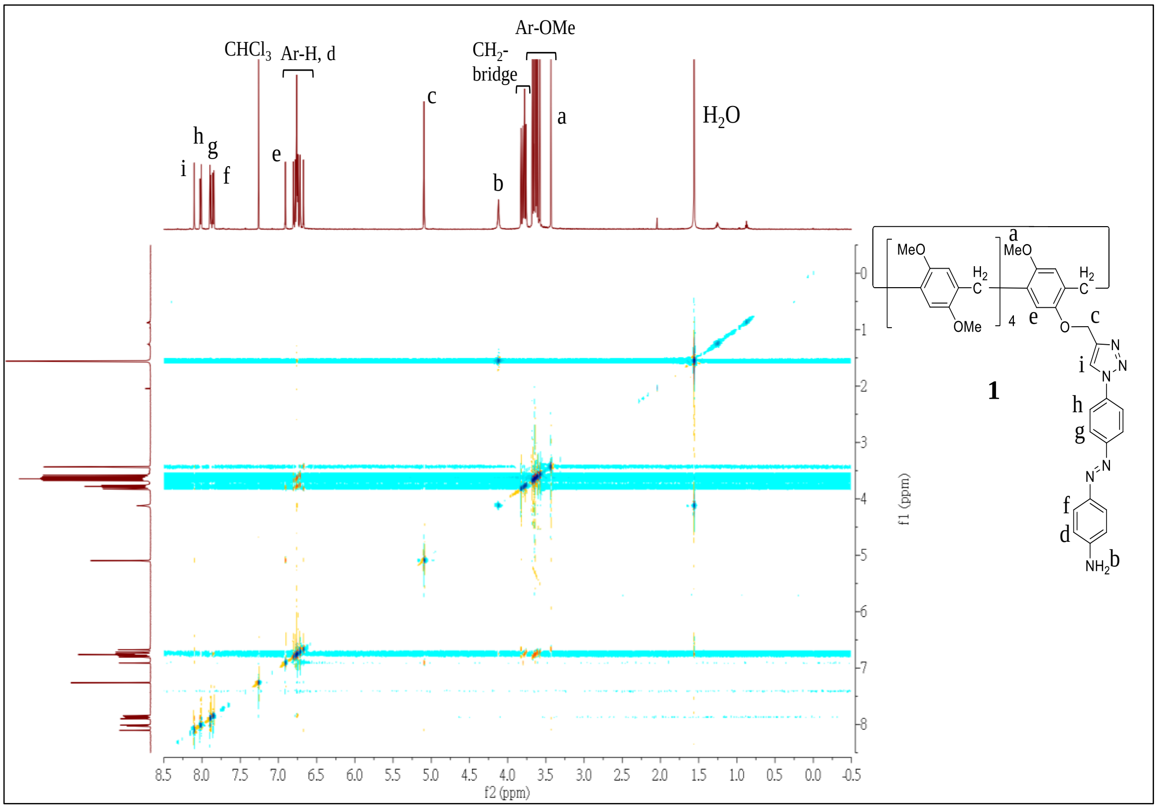

Figure S4. NOESY spectrum (600 MHz) of pillararene **1** (10 mM in $CDCl_3$).

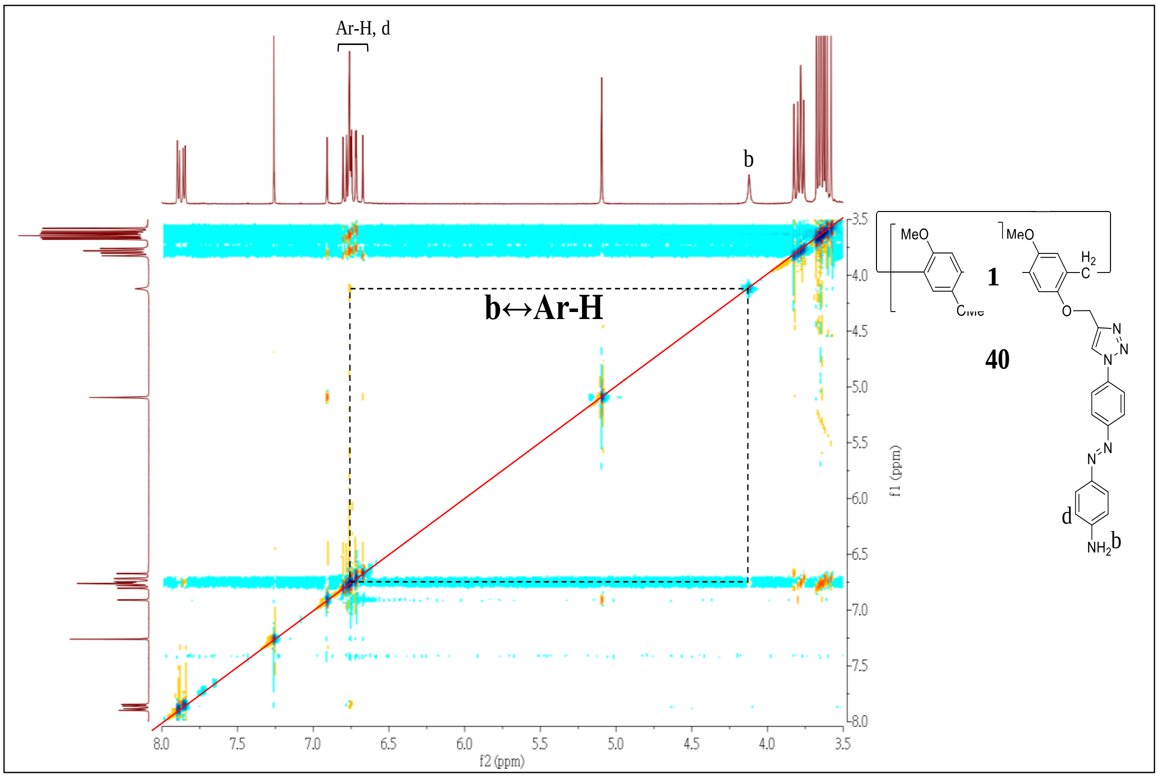

Figure S5. Partial NOESY spectrum (600 MHz) of pillararene **1** (10 mM in $CDCl_3$).

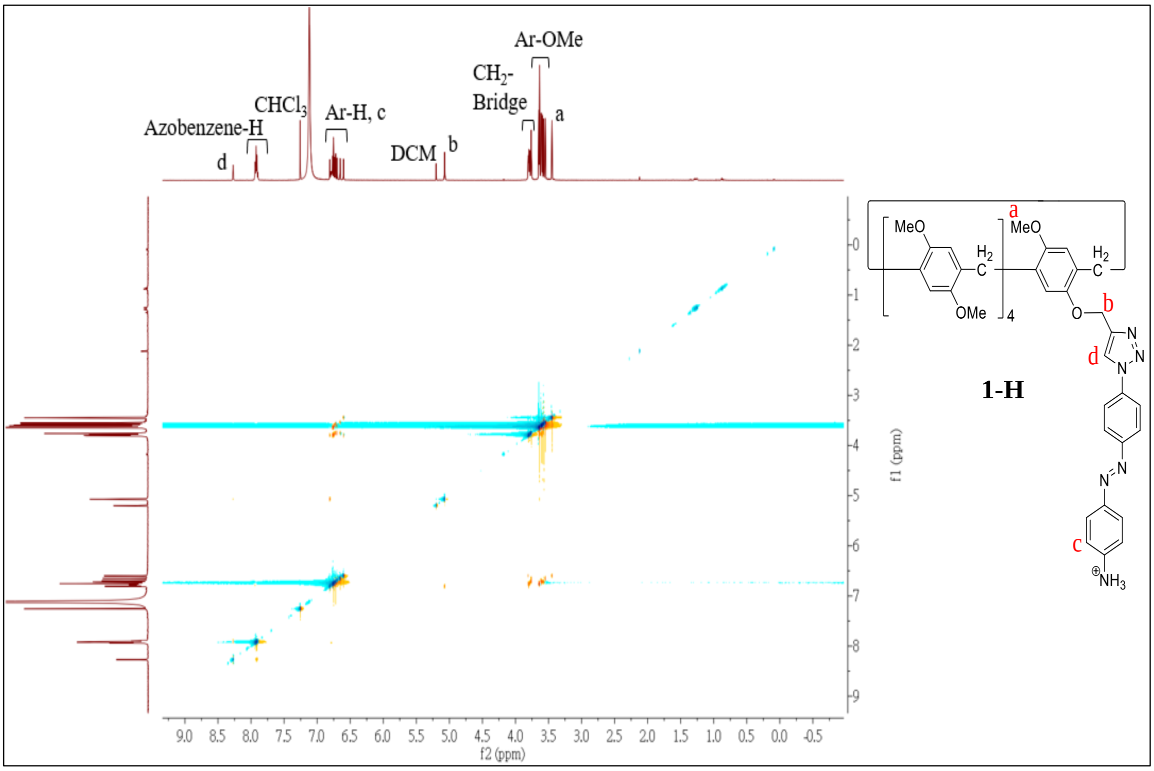

Figure S6. ROESY spectrum (600 MHz) of pillararene **1-H** (5 mM in $CDCl_3$).

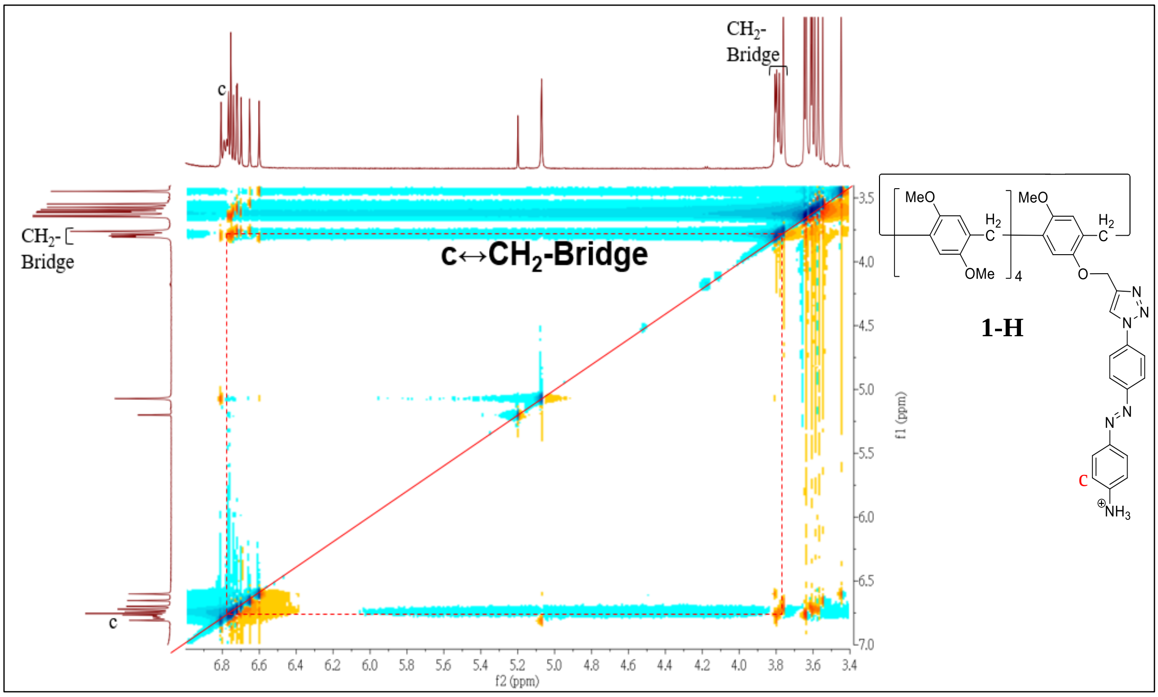

Figure S7. Partial ROESY spectrum (600 MHz) of pillararene **1-H** (5 mM in $CDCl_3$)

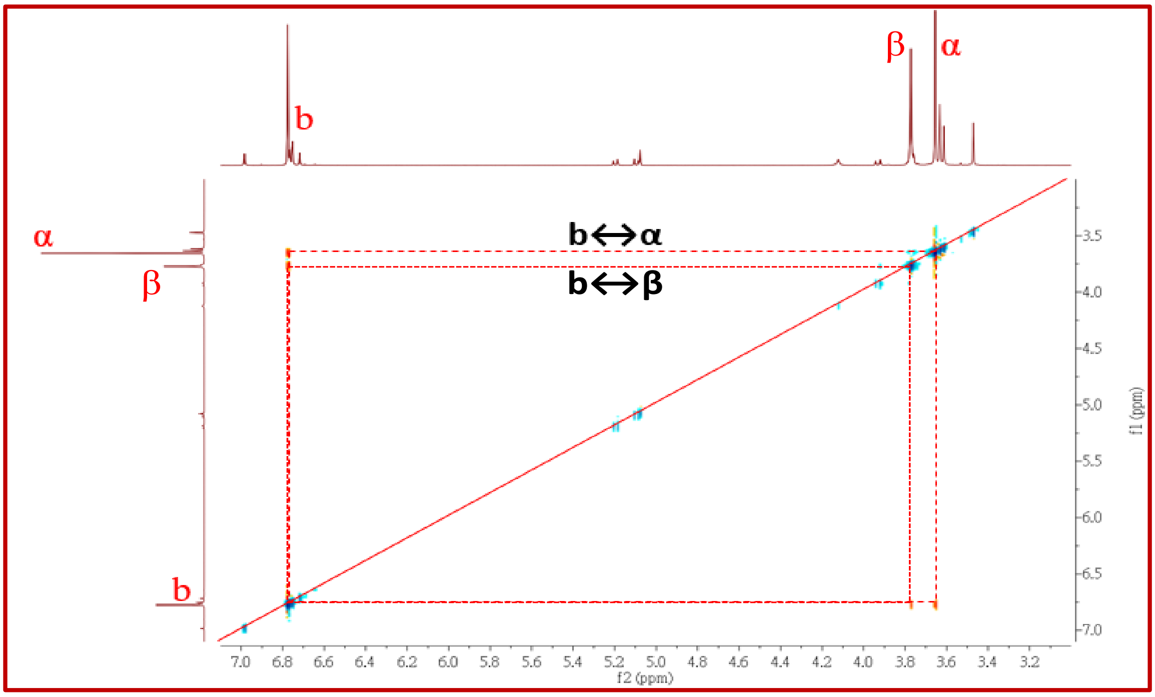

Figure S8. Partial NOESY spectrum (600 MHz) of a 1:1 molar mixture of pillararenes **2** and **DMP5** (5 mM in $CDCl_3$).

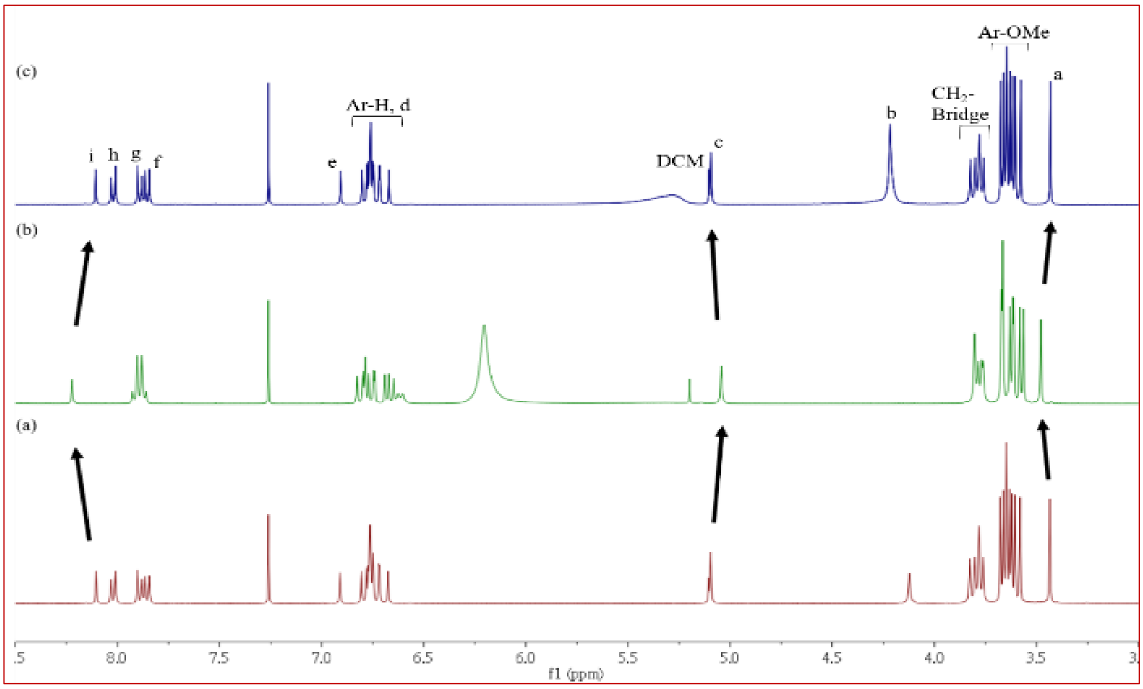

Figure S9. Partial ^{1}H NMR spectra (400 MHz) of (a) pillararene **1** (2.5 mM in $CDCl_3$), (b) obtained by adding excess of TFA to the solution (a), and (c) obtained by adding excess of K_2CO_3 to the solution (b).

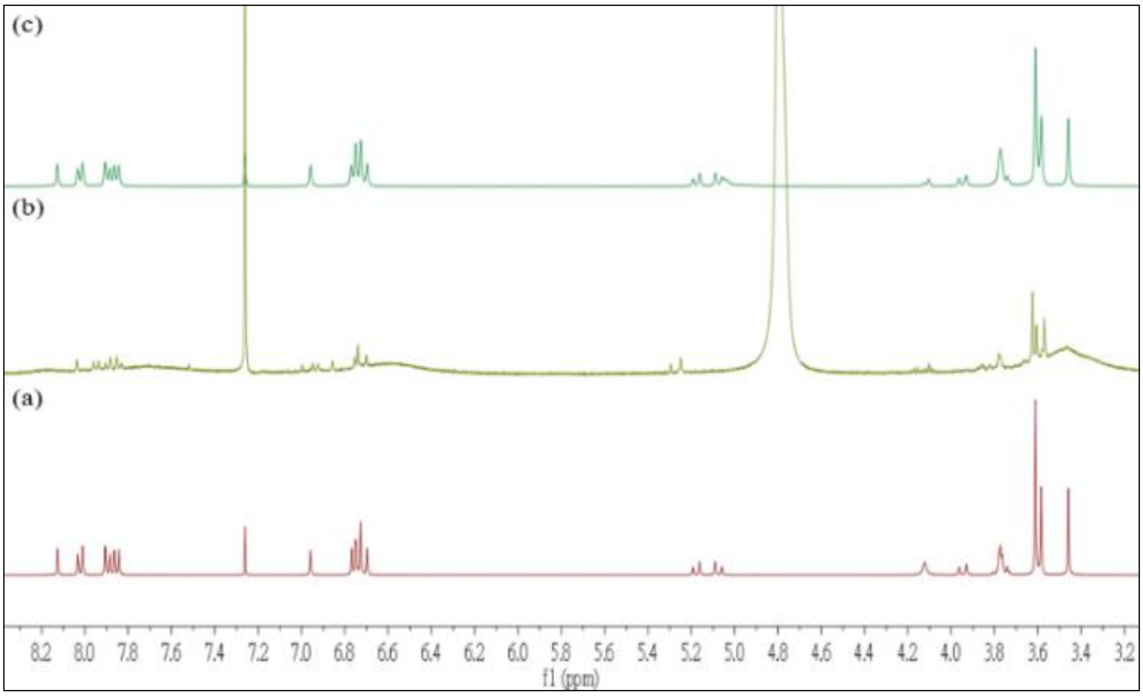

Figure S10. Partial ^1H NMR spectra (400 MHz, $CDCl_3$, 2.5 mM) of (a) pillararene **2**, (b) obtained by adding excess of TFA to the solution of (a), and (c) obtained by adding excess K_2CO_3 to the solution of (b).

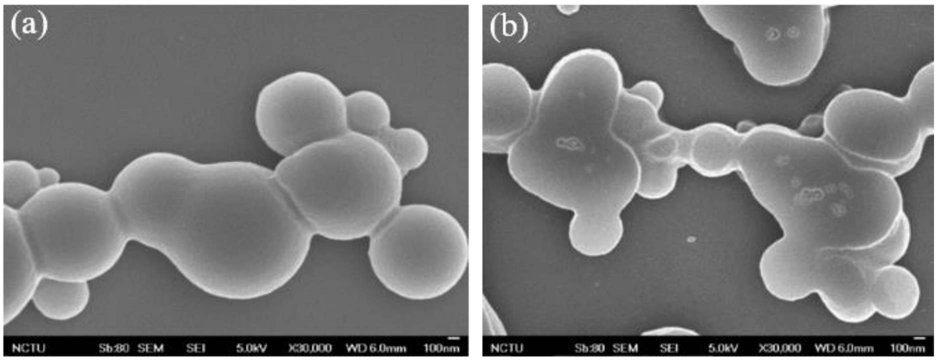

Figure S11. FE-SEM images of pillararene **1-H** (10^{-4} M, chloroform): (a) before and (b) after UV light (365 nm) irradiation for 1 h. Scale bar is 100 nm for both (a) and (b).

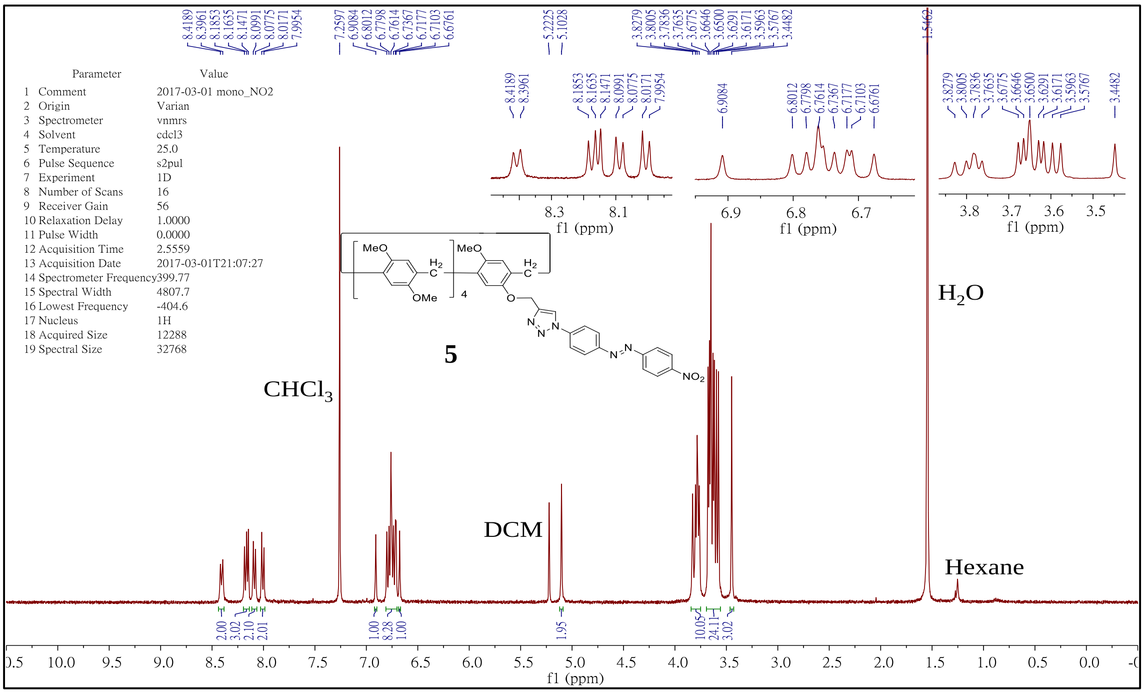

Figure S12. ^{1}H NMR (400 MHz, $CDCl_3$) spectrum of pillararene **5**.

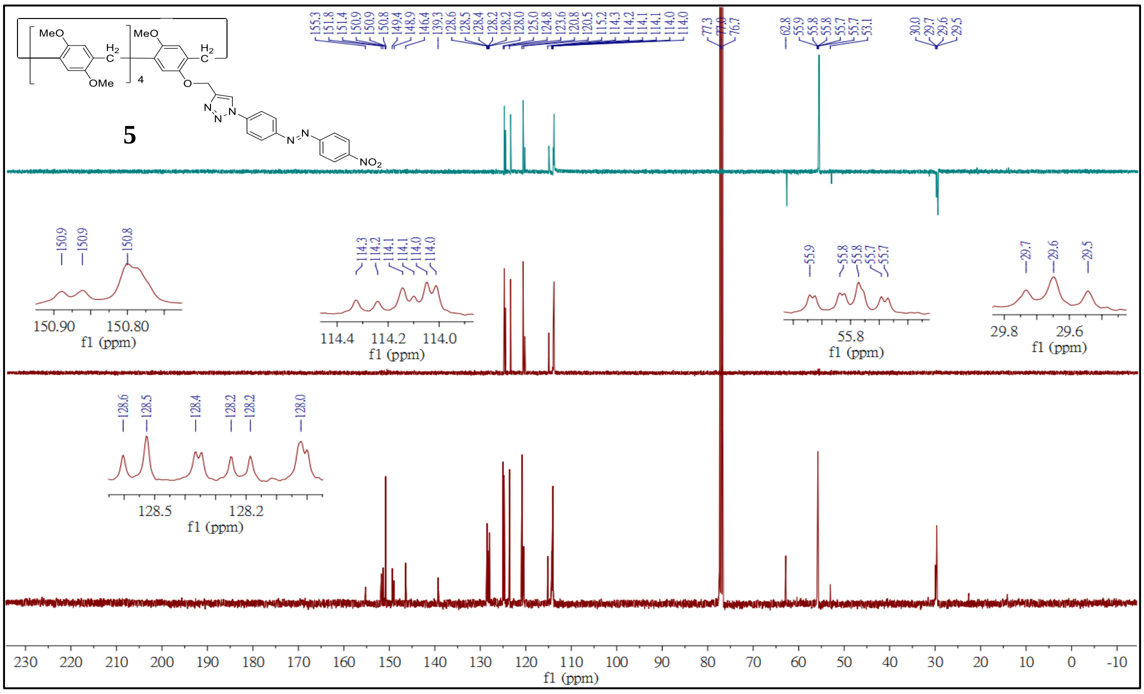

Figure S13. DEPT (100 MHz, $CDCl_3$) spectra of pillararene **5**.

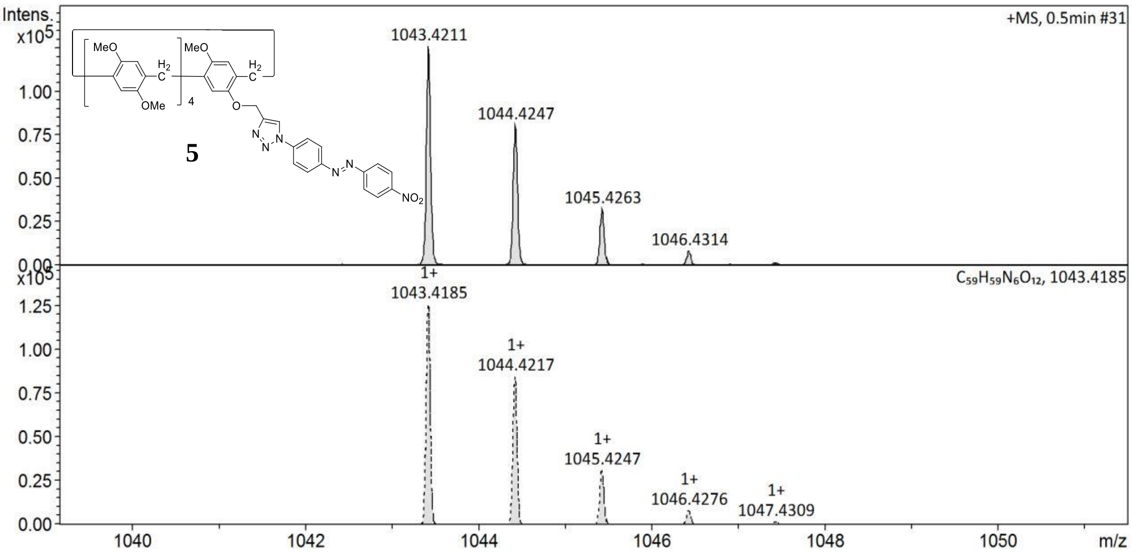

Display Report

Meas. m/z	#	Ion Formula	m/z	err [ppm]	mSigma	# Sigma	Score	rdb	e⁻ Conf	N-Rule	Adduct
1043.4211	1	C59H59N6O12	1043.4185	2.4	14.3	1	100.00	33.5	even	ok	M+H

Figure S14. HRMS-ESI (+) mass spectrometry of pillararene **5**.

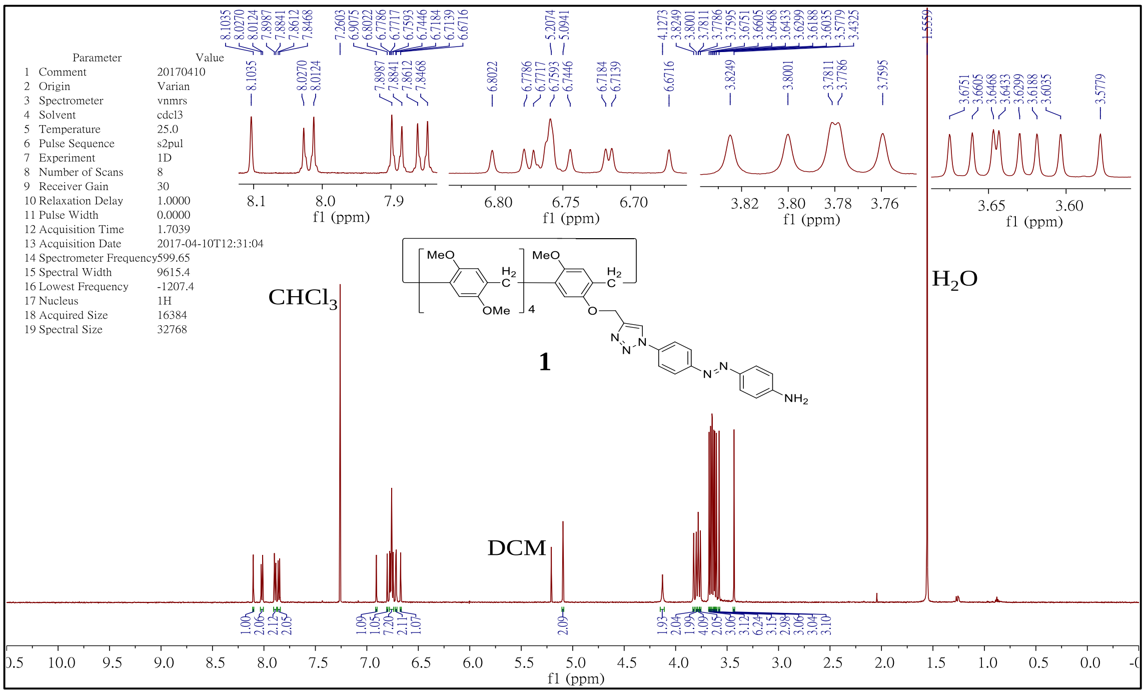

Figure S15. ^{1}H NMR (400 MHz, $CDCl_3$) spectrum of pillararene **1**.

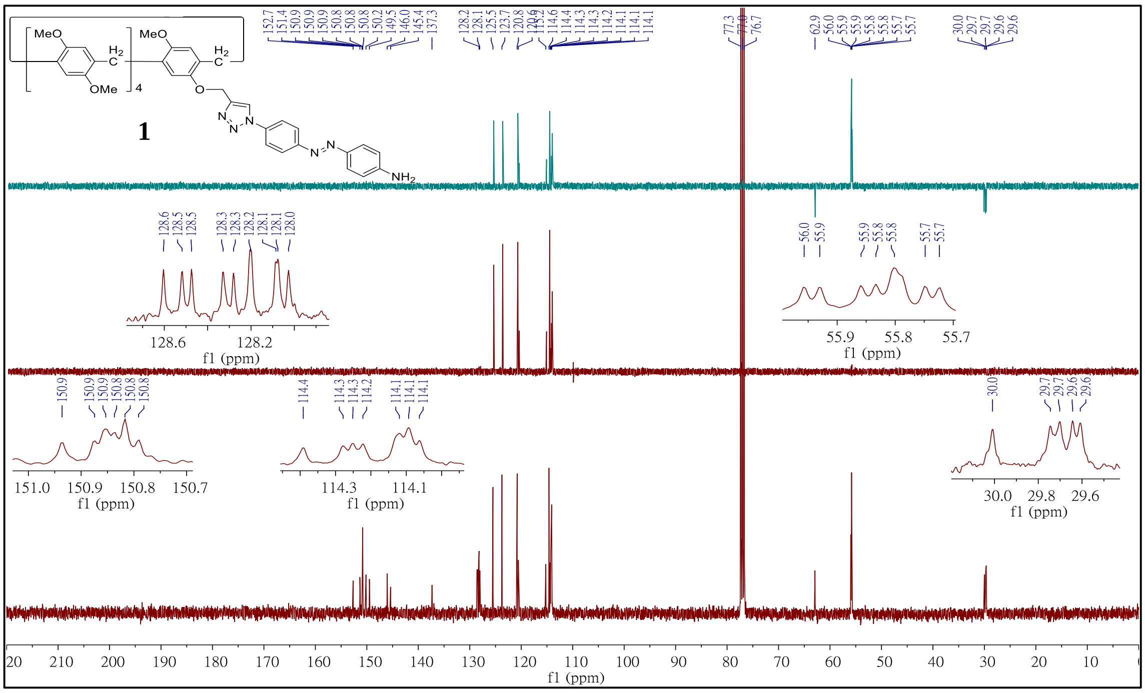

Figure S16. DEPT (100 MHz, $CDCl_3$) spectra of pillararene **1**.

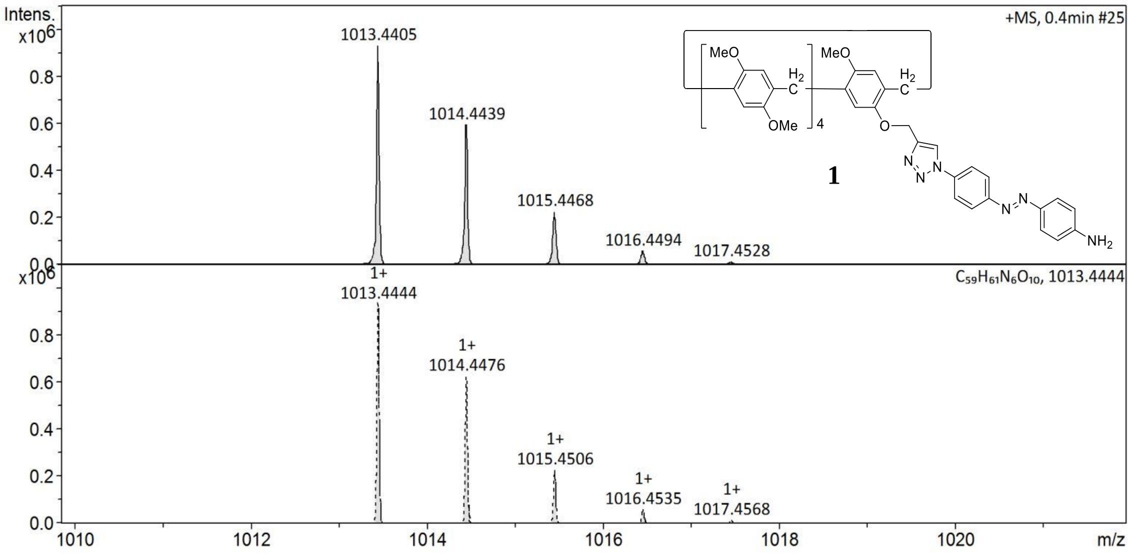

Display Report

Meas. m/z	#	Ion Formula	m/z	err [ppm]	mSigma	# Sigma	Score	rdb	e⁻ Conf	N-Rule	Adduct
1013.4405	1	C59H61N6O10	1013.4444	3.8	14.1	1	100.00	32.5	even	ok	M+H

Figure S17. HRMS-ESI (+) mass spectrometry of pillararene **1**.

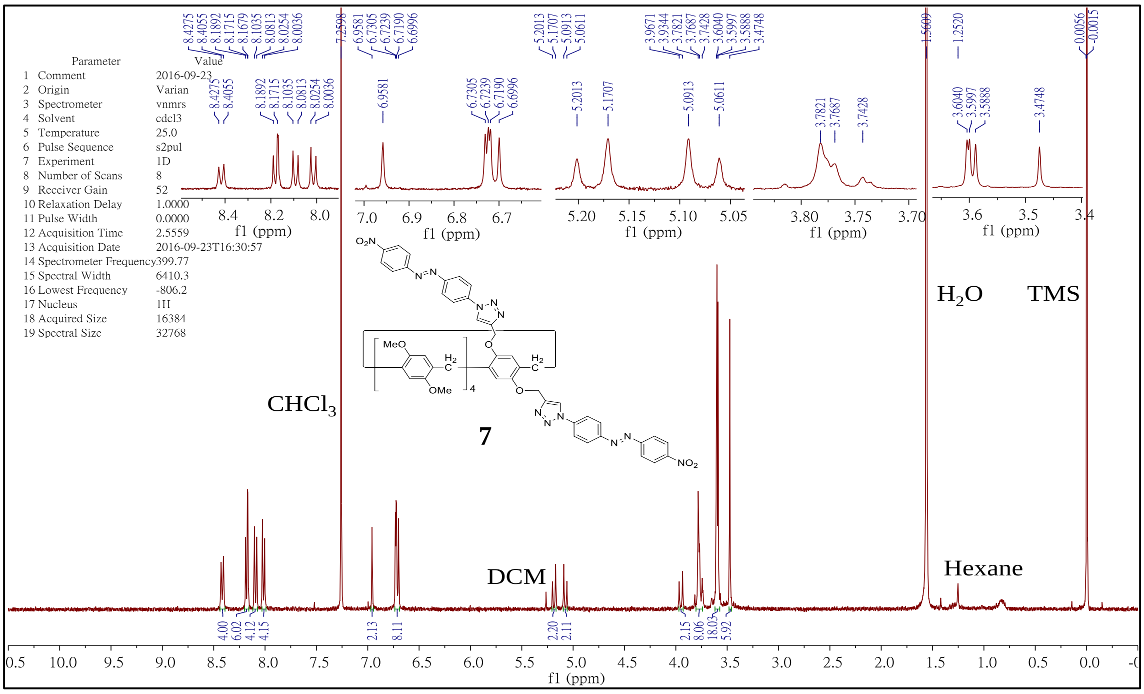

Figure S18. ^{1}H NMR (400 MHz, $CDCl_3$) spectrum of pillararene **7**.

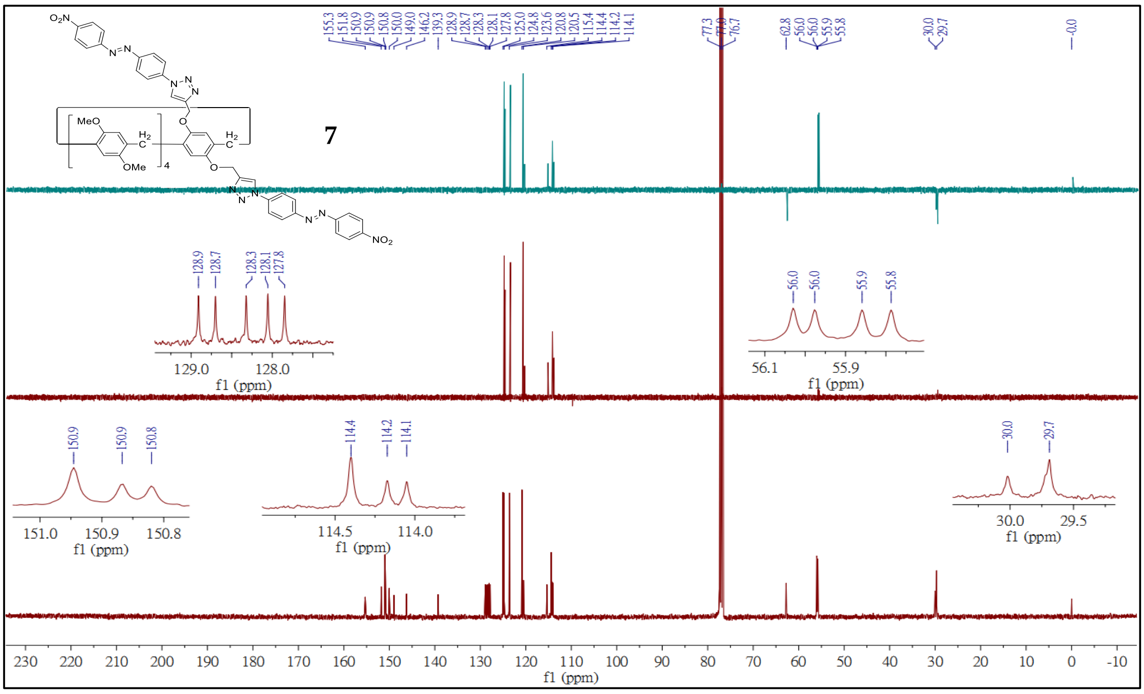

Figure S19. DEPT (100 MHz, $CDCl_3$) spectra of pillararene **7**.

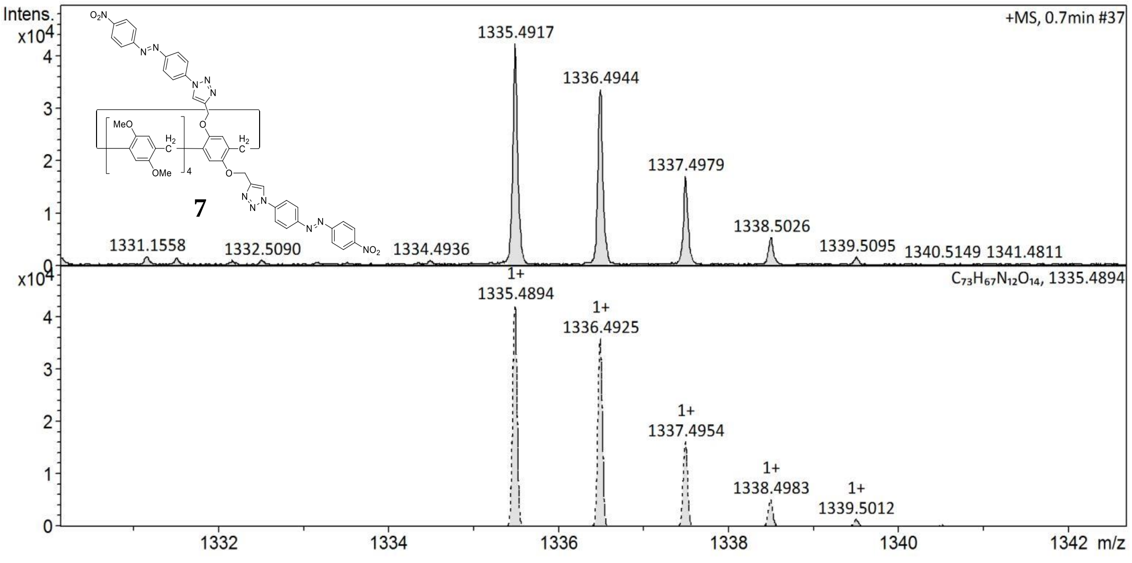

Display Report

Meas. m/z	#	Ion Formula	m/z	err [ppm]	mSigma	# Sigma	Score	rdb	e⁻ Conf	N-Rule	Adduct
1335.4917	1	C73H67N12O14	1335.4894	1.7	23.4	1	100.00	46.5	even	ok	M+H

Figure S20. HRMS-ESI (+) mass spectrometry of pillararene **7**.

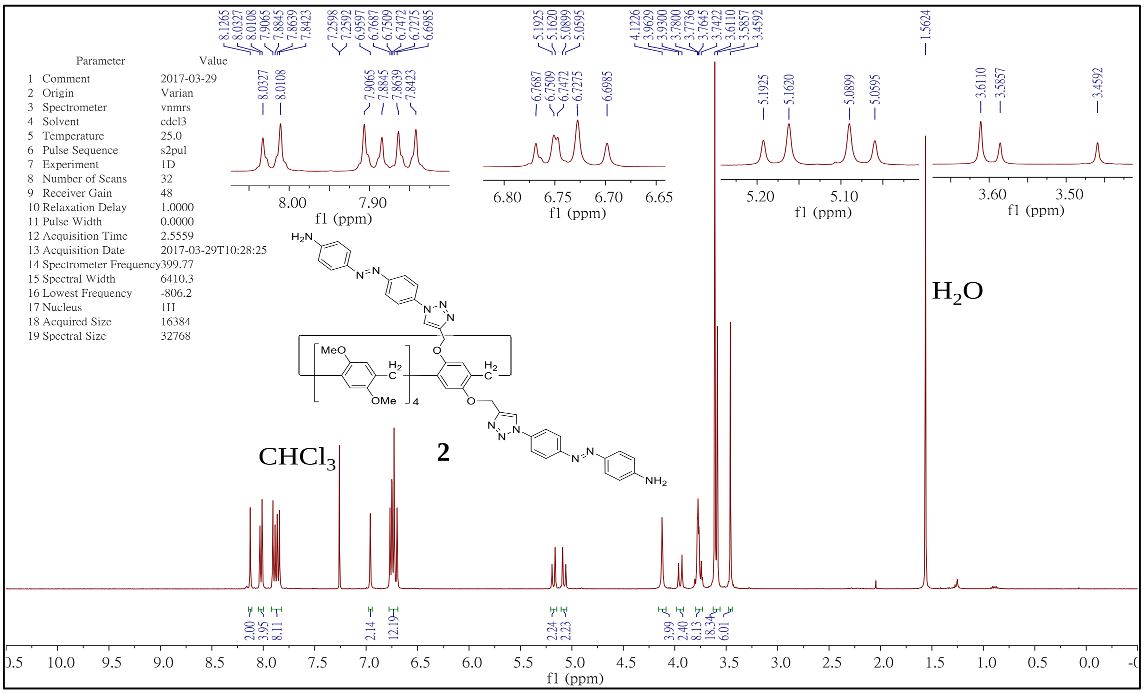

Figure S21. ^{1}H NMR (400 MHz, $CDCl_3$) spectrum of pillararene **2**.

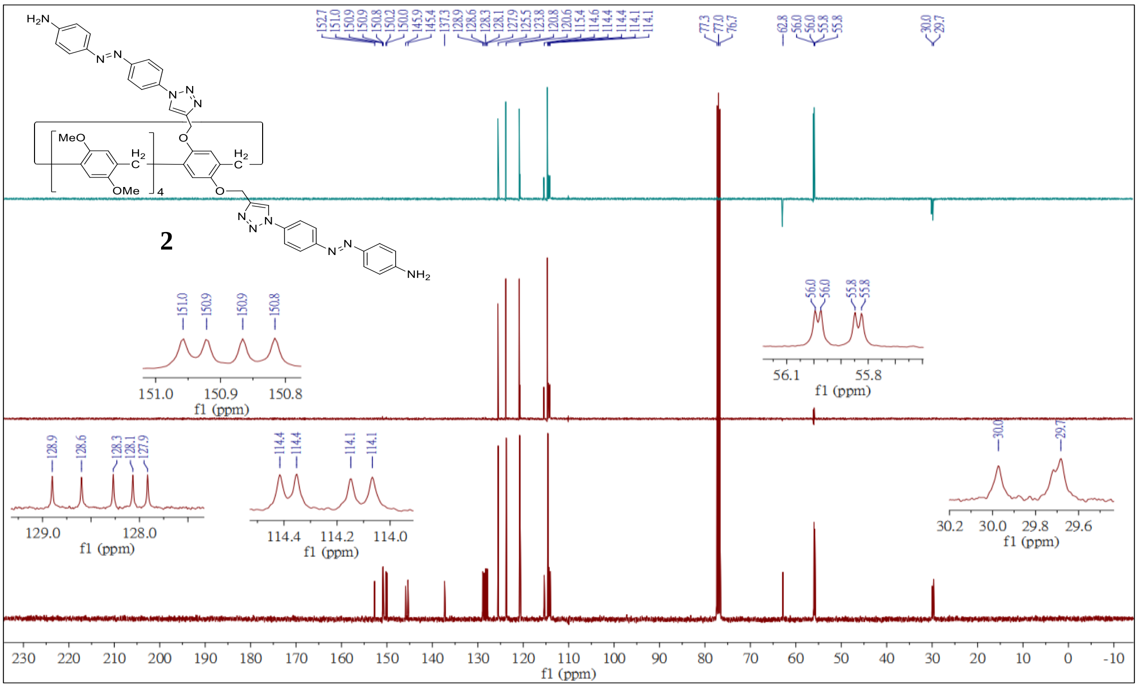

Figure S22. DEPT (100 MHz, $CDCl_3$) spectra of pillararene **2**.

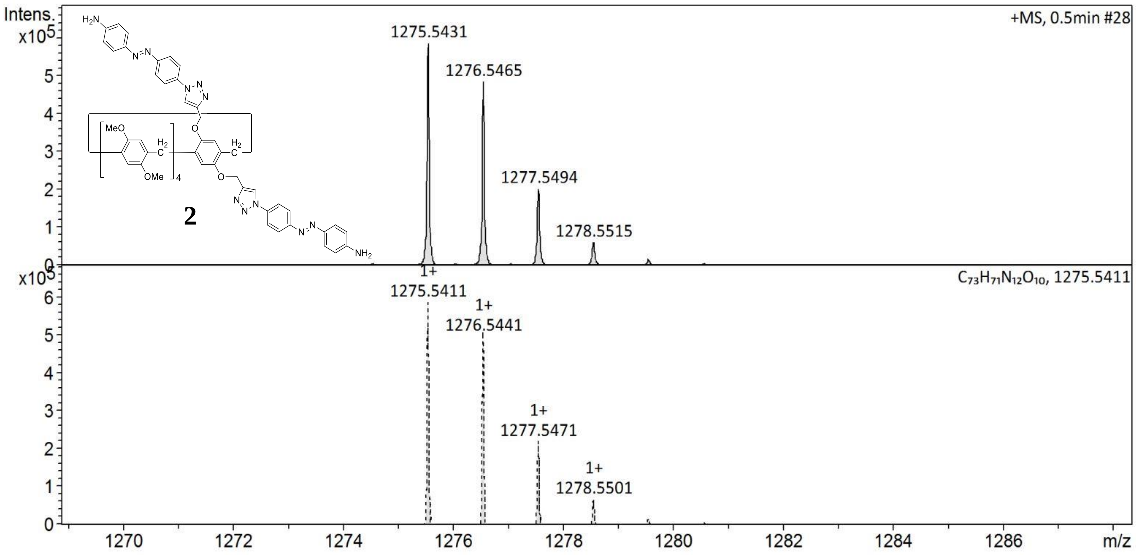

Display Report

Meas. m/z	#	Ion Formula	m/z	err [ppm]	mSigma	# Sigma	Score	rdb	e⁻ Conf	N-Rule	Adduct
1275.5431	1	C73H71N12O10	1275.5411	-1.6	14.7	1	100.00	44.5	even	ok	M+H

Figure S23. HRMS-ESI (+) mass spectrometry of pillararene **2**.

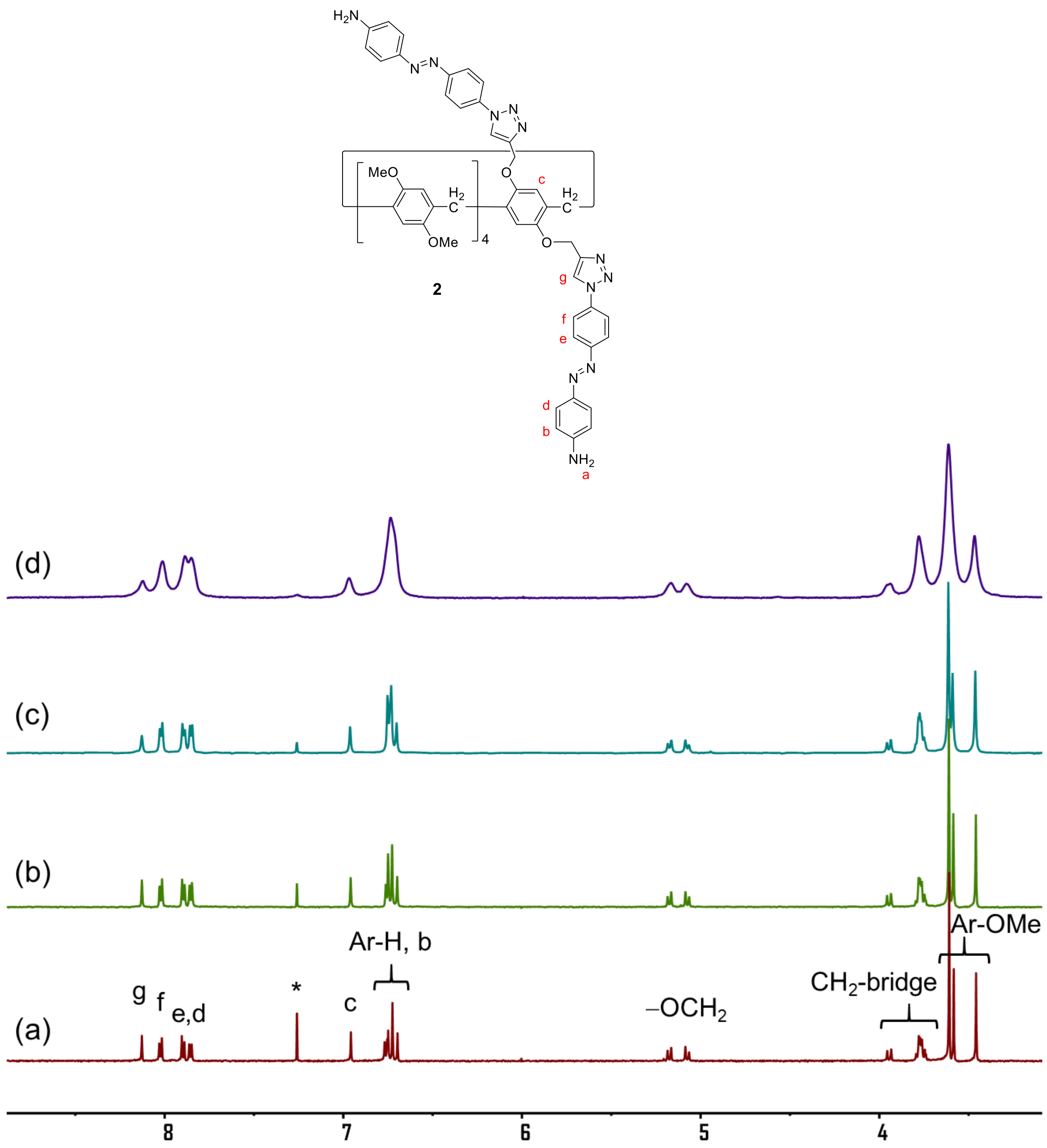

Figure S24. ^{1}H **NMR** spectra (600 MHz, 298 K) of pillararenes **2** in $CDCl_3$ at various concentrations: (a) 2.5 mM, (b) 5 mM, (c) 10 mM, and (d) 25 mM, where * denotes the residual signal of $CHCl_3$ in $CDCl_3$.

Table S1. Concentration dependence of the diffusion coefficient *D* (600 MHz, $CDCl_3$, 298K) of pillararenes **1**, **1-H**, **2**, and 1:1 molar mixture of pillararenes **2** and **DMP5**.

Concentration (mM)	D (10^{-10} m^2/s) pillararene **1**	D (10^{-10} m^2/s) pillararene **1-H**	D (10^{-10} m^2/s) pillararene **2**	D (10^{-10} m^2/s) 1:1 (**2** + **DMP5**)
2.5	8.32	6.18	6.89	7.05
5	7.91	5.60	6.87	6.73
10	7.37	5.06	6.86	6.13
25	6.70	4.22	6.88	5.38
50	6.11	3.53	–	4.42
75	6.02	3.20	–	4.25

(a) Monomer **1-H**

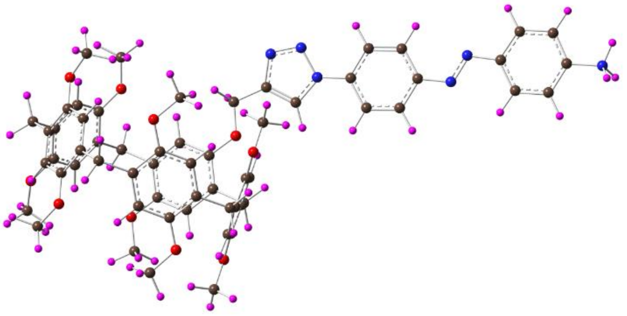

(b) Side view of (**1-H**)$_2$

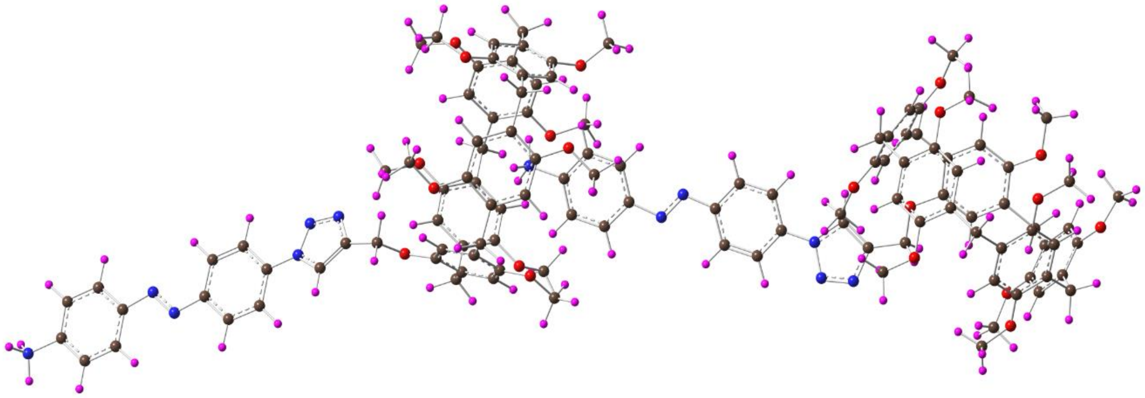

(c) Top view of (**1-H**)$_2$

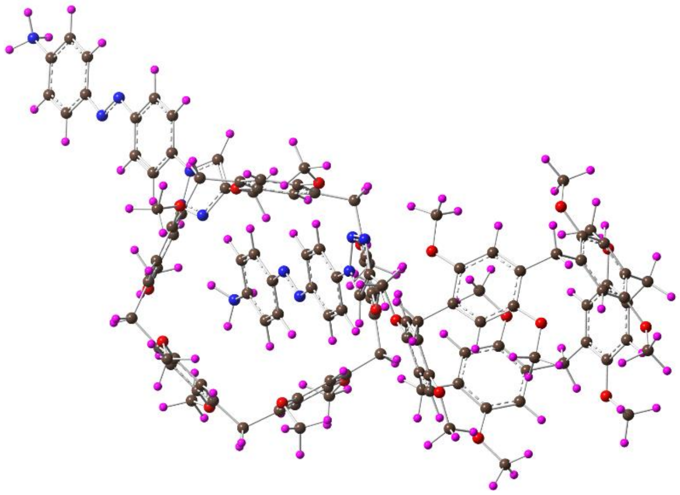

Figure S25: Geometry optimizations of (a) **1-H**, (b) side view of (**1-H**)$_2$, and (c) top view of (**1-H**)$_2$, computed using Gaussian 16 under B3LYP/6-31G(d,p) level in the gas phase.

(a) Monomer **2**

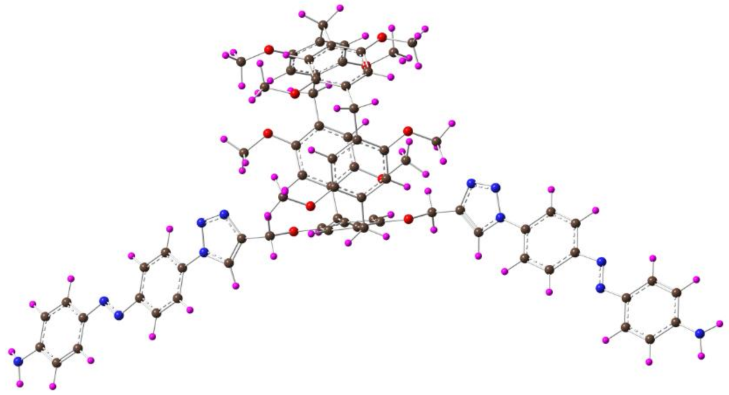

(b) Side view-1 of dimer **2**

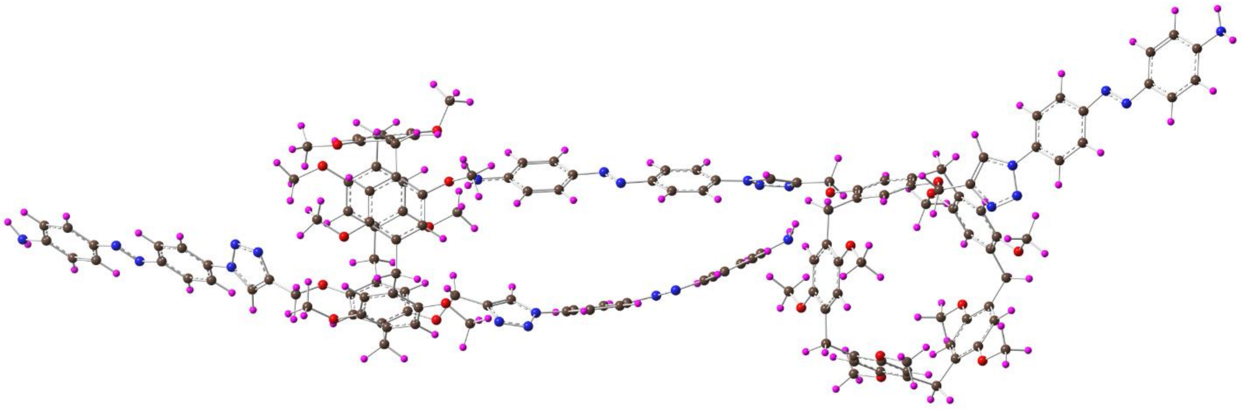

(c) Side view-2 of dimer **2**

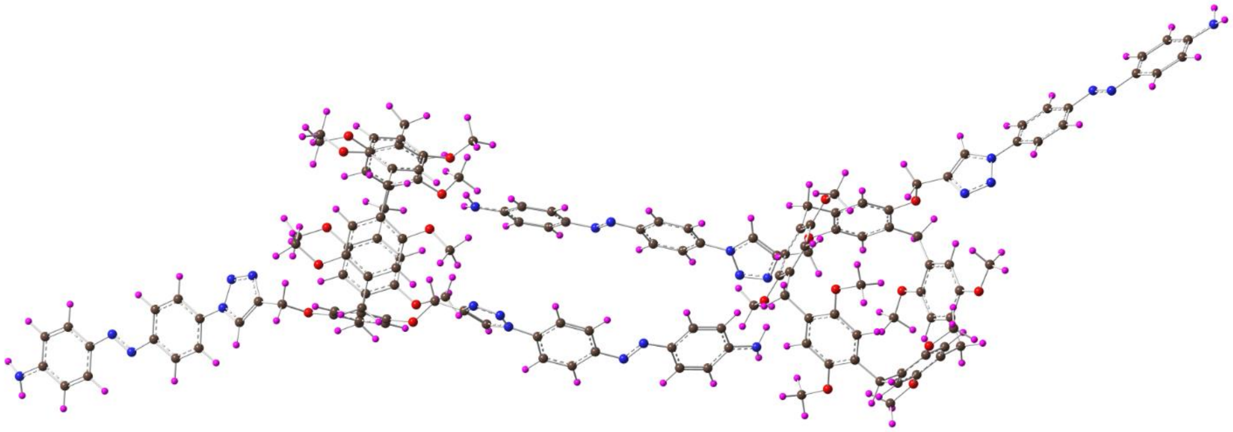

Figure S26: Geometry optimizations of (a) **2**, (b) side view-1 of dimer **2**, and (c) side view-2 of dimer **2**, computed using Gaussian 16 under B3LYP/6-31G(d,p) level in the gas phase.

Theoretical study

To further investigate the relative stability of the monomeric and dimeric forms of compounds **1** (neutral), **1–H** (charge = +1), and their dimers—namely, $\mathbf{1}_2$ and $(\mathbf{1–H})_2$ (charge = +2)—Density Functional Theory (DFT) calculations were performed using the B3LYP method[1] with the Gaussian 16 software package.[2] The geometries of all compounds were fully optimized at the B3LYP/6-31G(d,p) level, and the lowest-energy conformations are reported (vide infra).

The optimized geometries of compounds **1** (neutral), **1–H** (charge = +1), **2**, and the dimer of **2** (neutral), along with their corresponding structures, are shown in **Figures S25** and **S26**. As illustrated in **Figure S25**, the cationic monomer **1–H** forms a stable dimer in which the macrocycle is positioned around the central $–NH_3^+$ moiety of the thread via strong hydrogen-bonding interactions. The binding energy of this $–NH_3^+$ dimer, $(\mathbf{1–H})_2$, is calculated to be –22.1 kcal/mol. In this structure, the hydrogen bond distance between the protonated $–NH_3^+$ group and the benzene ring in the macrocyclic cavity is 2.26 Å .

In contrast, for the dimer of compound **2**, which contains two triazolylazoaniline groups attached to opposite side of the same copillar[5]arene, the macrocycle is displaced outward due to steric repulsion between the bulky triazolylazoaniline units from the second molecule of compound **2** (see **Figures S26b** and **S26c**). The binding energy of dimer **2** is calculated to be –6.5 kcal/mol. The Cartesian coordinates of the optimized structures are provided in **Tables S2–S5**.

References:

1. Becke AD (1993) Density-functional thermochemistry. III. The role of exact exchange. J Chem Phys 98:5648-5652

2. Frisch MJ, Trucks GW, Schlegel HB, Scuseria GE, Robb MA, Cheeseman JR, Scalmani G, Barone V, Petersson GA, Nakatsuji H, Li X, Caricato M, Marenich AV, Bloino J, Janesko BG, Gomperts R, Mennucci B, Hratchian HP, Ortiz JV, Izmaylov AF, Sonnenberg JL, Williams, Ding F, Lipparini F, Egidi F, Goings J, Peng B, Petrone A, Henderson T, Ranasinghe D, Zakrzewski VG, Gao J, Rega N, Zheng G, Liang W, Hada M, Ehara M, Toyota K, Fukuda R, Hasegawa J, Ishida M, Nakajima T, Honda Y, Kitao O, Nakai H, Vreven T, Throssell K, Montgomery Jr. JA, Peralta JE, Ogliaro F, Bearpark MJ, Heyd JJ, Brothers EN, Kudin KN, Staroverov VN, Keith TA, Kobayashi R, Normand J, Raghavachari K, Rendell AP, Burant JC, Iyengar SS, Tomasi J, Cossi M, Millam JM, Klene M, Adamo C, Cammi R, Ochterski JW, Martin RL, Morokuma K, Farkas O, Foresman JB, Fox DJ (2016) Gaussian 16, Revision C.01. Gaussian Inc: CT.

Table S2: Optimized Cartesian structures of monomer **1-H** at the B3LYP/6-31G (d,p) level.

**Monomer 1-H (Charge = +1)**							
C	-6.77005500	2.15782300	-1.11568500	C	-0.52134400	4.26351900	1.44421500
C	-5.86343600	2.72303800	-2.02693500	H	0.31086200	4.67846600	0.86975800
C	-5.38896400	1.92264400	-3.07017000	H	-0.66942700	4.89718200	2.32249000
H	-4.68167200	2.36519000	-3.76046800	C	-1.77752500	4.29428700	0.58710700
C	-5.78302200	0.58881800	-3.20560700	C	-1.69349500	4.19777900	-0.80494900
C	-6.67877200	0.01946700	-2.28622900	H	-0.70738900	4.11808600	-1.24474600
C	-7.16697500	0.82504200	-1.25346400	C	-2.83794600	4.17123500	-1.60634800
H	-7.84766000	0.37225500	-0.54339700	C	-4.11266300	4.24349600	-1.02159500
C	-8.21829000	2.48737000	0.76496700	C	-4.19568600	4.36137000	0.36866900
H	-8.46978500	3.30906100	1.43832700	H	-5.18381900	4.40467900	0.80928500
H	-9.12030000	2.18050700	0.21995500	C	-3.05140600	4.38765700	1.17040500
H	-7.85572100	1.63573700	1.35568300	C	-5.37414400	4.15414800	-1.86639200
C	-4.51490100	0.32586700	-5.22176500	H	-6.15978200	4.75198200	-1.39812100
H	-3.55866400	0.68416200	-4.81772600	H	-5.17580100	4.57689600	-2.85423000
H	-4.32285700	-0.47365700	-5.93995400	C	-4.34855300	4.68328000	3.16311100
H	-5.01475600	1.15755400	-5.73511700	H	-4.85776300	5.58297300	2.79436700
C	-6.43726500	-2.65267400	-0.22941700	H	-5.00553900	3.81726900	3.00988500
C	-6.16260300	-2.34973700	-1.57306700	C	-1.53783600	4.08894300	-3.61769900
C	-4.99877800	-2.87244700	-2.14407800	H	-0.92627900	3.22122500	-3.33572800
H	-4.79172400	-2.62267500	-3.17720200	H	-1.73729600	4.05183600	-4.69039400
C	-4.11282400	-3.66144900	-1.40575500	O	-7.22160500	2.98545800	-0.10939400
C	-4.37894900	-3.95020400	-0.05769400	O	-5.33507900	-0.23766800	-4.21473700
C	-5.55221700	-3.44334100	0.50841100	O	-7.60169000	-2.12878200	0.28924600
H	-5.74363400	-3.66393400	1.55097400	O	-2.95259100	-4.19504100	-1.92775300
C	-7.96821100	-2.49337200	1.60755200	O	-3.72516100	-3.58202200	3.30060600
H	-7.24079100	-2.13501500	2.34807900	O	1.06523200	-2.55883500	0.69420400
H	-8.93349700	-2.01986300	1.79627000	O	-0.84021300	0.52586300	4.72162800
H	-8.07454300	-3.58076100	1.71388700	O	1.09297700	2.54306200	-0.07241700

C	-2.70342900	-4.01759200	-3.31122100	O	-3.08700700	4.50366900	2.54375300
H	-2.57067000	-2.95895100	-3.57010100	O	-2.80138800	4.07278600	-2.98123000
H	-1.77895100	-4.55739600	-3.52564400	H	-0.97782300	5.00507200	-3.38800600
H	-3.51251300	-4.43267400	-3.92576000	H	-4.14947200	4.79813500	4.23041700
C	-7.08024500	-1.44448600	-2.38008200	C	2.73937900	-2.83941900	-0.91935100
H	-8.10498100	-1.55765800	-2.01822100	C	3.72409700	-2.24812900	-0.17221600
H	-7.05862400	-1.75651200	-3.42697400	N	3.23698100	-3.09333300	-2.17358200
C	-3.40343600	-4.76309000	0.77935800	H	3.73345600	-1.91496300	0.85052900
H	-3.95684400	-5.29033100	1.56025700	N	4.46562000	-2.69834200	-2.24208500
H	-2.92329300	-5.51310600	0.14560800	N	4.79731700	-2.16882700	-1.01986600
C	-0.14609200	-2.82572000	1.31701300	C	6.08234100	-1.64076200	-0.80527400
C	-1.12216900	-3.65720300	0.76422000	C	7.08133100	-1.85296500	-1.77867800
H	-0.98592300	-4.11123400	-0.20934400	C	6.36794000	-0.91238600	0.35901400
C	-2.32935700	-3.90107000	1.42388900	C	8.34909100	-1.34301300	-1.58396500
C	-2.53328300	-3.29798600	2.67728200	H	6.83208200	-2.41677800	-2.66820700
C	-1.55597800	-2.46273200	3.22490200	C	7.64530400	-0.40748800	0.54956500
H	-1.71617200	-1.97014000	4.17590600	H	5.60095600	-0.73031700	1.10179600
C	-0.35559100	-2.20404000	2.55562100	C	8.64859800	-0.61372900	-0.41248100
C	-3.95469600	-3.03644000	4.58812600	H	9.12678400	-1.49944700	-2.32166200
H	-3.97661000	-1.93918700	4.56732800	H	7.89017000	0.16028400	1.44064300
H	-4.93029400	-3.40902500	4.90513000	N	9.89348100	-0.05676900	-0.11195400
H	-3.19412600	-3.36320700	5.30902500	N	10.80277600	-0.23938600	-0.97727100
C	1.34496500	-3.21154300	-0.52878500	C	12.04098500	0.34369400	-0.62350000
H	0.64417200	-2.91105600	-1.32035600	C	12.27947800	1.06391100	0.56384800
H	1.26342600	-4.30344800	-0.42009900	C	13.07795600	0.17058100	-1.55294500
C	0.65994700	-1.23745100	3.14470300	C	13.53474000	1.59970300	0.81558100
H	1.66349100	-1.52738600	2.82128000	H	11.46617000	1.19070700	1.26787900
H	0.63122000	-1.31133300	4.23419600	C	14.34189300	0.70225400	-1.31341600
C	-0.37516300	1.05688400	3.53811300	H	12.87044400	-0.38383500	-2.46116300
C	0.40049800	0.20500100	2.73630800	C	14.53905800	1.40755700	-0.13234700
C	0.89605400	0.70495500	1.52763000	H	13.71922400	2.15559200	1.73164000
H	1.47416000	0.03413600	0.90417600	H	15.14109500	0.56400800	-2.03707700
C	0.62367900	2.00969100	1.10932000	N	15.89845100	1.96537400	0.14966800
C	-0.16207400	2.85924100	1.90588500	H	16.42833500	1.38348000	0.81154200
C	-0.64233600	2.36453800	3.12114500	H	16.45234200	2.03194300	-0.71120700
H	-1.26235400	3.02078600	3.71838000	H	15.84084800	2.90950300	0.54878800
C	-1.58701300	1.36773900	5.58306200				
H	-2.51735000	1.71010900	5.11198700				
H	-1.82908200	0.76518100	6.46041300				
H	-1.00643600	2.24435800	5.89837400				
C	1.83833700	1.70431300	-0.93255600				
H	1.25952700	0.82838900	-1.25287900				
H	2.08872800	2.30878400	-1.80656500				
H	2.76886100	1.35718800	-0.46166100				

Table S3: Optimized Cartesian structures of the Dimer (**1-H**)$_2$ at the B3LYP/6-31G (d,p) level.

Dimer **(1-H)$_2$ (Charge = +2)**	
C -4.43294500 4.91940300 1.73880200	H -26.12437900 -5.91820700 -1.40935500
C -5.66921000 5.56860300 1.80705300	N -5.98582100 1.91763300 -1.09303900
C -6.51860900 5.25270600 2.87786300	C -4.59504400 1.50170900 -1.40584800
H -7.48020500 5.74792300 2.92722100	C -3.53806600 2.29397400 -0.95717600
C -6.14946200 4.31387400 3.84451000	C -4.39932200 0.32434600 -2.12063900
C -4.90849000 3.64599900 3.76032500	C -2.23829100 1.89029500 -1.24440000
C -4.06761200 3.97150300 2.69826900	H -3.70506900 3.21286300 -0.40006100
H -3.11636700 3.45677300 2.60853700	C -3.09110700 -0.06435400 -2.40102700
C -2.38737200 5.88752300 0.99714300	H -5.23786400 -0.27951000 -2.45578700
H -1.82699000 6.01508500 0.06880300	C -2.00508400 0.70870700 -1.96852800
H -2.62334300 6.87083000 1.41994500	H -1.39024900 2.47991600 -0.91801300
H -1.77766000 5.32935800 1.71621000	H -2.88669300 -0.96901600 -2.96335000
C -8.13354000 4.71559200 5.13017100	N -0.72386200 0.20913500 -2.31745000
H -8.85849800 4.57002500 4.31910800	N 0.23415400 0.93635400 -1.92863900
H -8.55576600 4.33576300 6.06140400	C 1.51844400 0.46848800 -2.25806700
H -7.92610800 5.78691300 5.23781400	C 1.78797300 -0.72262000 -2.96138200
C -4.13165500 0.38275600 3.53425100	C 2.58372500 1.27680700 -1.83502000
C -4.95739600 1.18618000 4.33958500	C 3.09254900 -1.08660700 -3.23877100
C -6.20450400 0.68324700 4.72063500	H 0.96001100 -1.34098200 -3.28653000
H -6.83491300 1.31265600 5.33645700	C 3.89758200 0.91457100 -2.10262900
C -6.63856100 -0.58163200 4.31286100	H 2.35913000 2.18604700 -1.28786900
C -5.81632800 -1.38179200 3.50248800	C 4.15383300 -0.26618700 -2.81084300
C -4.56558100 -0.88192800 3.12612300	H 3.31984700 -1.99504800 -3.78182200
H -3.94076000 -1.50578000 2.49838900	H 4.71189600 1.53602800 -1.74942200
C -1.96257800 0.06663300 2.55937700	N 5.47875200 -0.65059600 -3.10607700
H -2.28728500 -0.23343700 1.55450500	C 6.63371400 0.08065600 -3.06197900
H -1.04293700 0.64809600 2.47711200	N 5.75222300 -1.93398600 -3.49549100
H -1.76882100 -0.83325900 3.15545600	C 7.61377000 -0.80313600 -3.43774100
C -8.62729100 -0.43842800 5.64604900	H 6.67652500 1.12433100 -2.80360200
H -8.97655500 0.54078200 5.29310400	N 7.03041400 -2.01776200 -3.68881400
H -9.49121800 -1.07434700 5.84652900	C 9.08133900 -0.57783000 -3.61554700
H -8.05843600 -0.30017100 6.57342100	H 9.63450300 -1.46756200 -3.28504700
C -4.51847100 2.57744300 4.76850800	H 9.31431100 -0.42509900 -4.68001100
H -3.43322000 2.58782200 4.89358900	O 9.42920800 0.56777100 -2.85898900
H -4.96940100 2.81334300 5.73486100	C 10.72775100 1.04424700 -2.95316500
C -6.28155400 -2.74469700 3.01444600	C 11.71848700 0.42183200 -3.71550000
H -5.41995100 -3.41001000 2.92863200	C 11.01400700 2.20878400 -2.22612600
H -6.96633500 -3.17427800 3.74912900	H 11.51807100 -0.49828600 -4.25003300
C -9.00535300 -2.23235900 0.37455700	C 13.01489800 0.93843600 -3.78652600
C -8.34915800 -2.37191000 1.60001800	C 12.30637800 2.73565500 -2.31128300
H -8.87844100 -2.24151800 2.53585800	C 9.97467800 2.86195600 -1.32838500
C -6.98270600 -2.65821400 1.66896900	C 14.09337700 0.21778700 -4.58022800
C -6.27541800 -2.82618800 0.46580700	C 13.29861000 2.11886100 -3.07810600
C -6.92933500 -2.66508900 -0.76104300	H 12.52301800 3.62846100 -1.73810000
H -6.38787800 -2.77997500 -1.69292700	H 8.97843400 2.69055200 -1.74486600
C -8.29163000 -2.35329700 -0.82909700	H 10.14531500 3.94084700 -1.31230900
C -4.25242800 -3.59238600 -0.57232600	C 10.01807500 2.32811900 0.09552100
H -4.12172300 -2.79222700 -1.31225900	C 14.86453100 -0.78488300 -3.73646400
H -3.26990800 -3.91910100 -0.22862100	H 14.79185700 0.95331400 -4.98642100
H -4.77107500 -4.43575400 -1.04406800	H 13.62842800 -0.30203000 -5.42158400
C -11.14204000 -1.96410700 1.44816500	O 14.58078300 2.60213500 -3.19147500
H -10.83507800 -1.13392800 2.09718600	C 10.80054400 2.95261800 1.08001500

H	-11.00695900	-2.90025200	2.00448400	C	9.31019300	1.17422600	0.44639300
C	-8.96928300	-2.11247400	-2.16933300	C	14.41630400	-2.10967200	-3.59233400
H	-10.00860100	-2.44504200	-2.11470500	C	16.02385500	-0.40158300	-3.05783900
H	-8.46674400	-2.70198000	-2.93892200	C	14.89423800	3.82725000	-2.55106800
C	-7.92276000	-0.12783800	-3.38725300	C	10.86661100	2.41678400	2.36963600
C	-8.94305500	-0.64478700	-2.57033700	O	11.47553200	4.08942400	0.69290600
C	-9.93272500	0.23509700	-2.11718500	H	8.72462000	0.68866000	-0.32440400
H	-10.71701000	-0.16772200	-1.48844900	C	9.38204500	0.63579400	1.73360900
C	-9.91817800	1.59629700	-2.44286100	C	15.11461600	-3.00428700	-2.77669800
C	-8.88095500	2.12210300	-3.23754200	O	13.27910200	-2.44720800	-4.29148700
C	-7.88999800	1.24130000	-3.69711100	C	16.72002400	-1.29500400	-2.23850600
H	-7.11425600	1.63919600	-4.34296700	H	16.35528600	0.62321800	-3.16718700
C	-6.34538000	-0.74020000	-5.09494100	H	14.79342100	3.75399800	-1.46050400
H	-5.60228400	0.06313900	-5.02159800	H	15.93435900	4.04236100	-2.80182300
H	-5.84101000	-1.66151600	-5.39019500	H	14.26064000	4.64733100	-2.91244700
H	-7.09370300	-0.47345700	-5.84851500	C	10.17348500	1.25396600	2.71611500
C	-12.05897600	1.97081200	-1.42143100	H	11.48645000	2.87566300	3.12939800
H	-11.85743500	1.48944600	-0.45997000	C	12.23090600	4.78344400	1.67106500
H	-12.70846900	2.83344400	-1.26788500	O	8.70345600	-0.49961500	2.12203600
H	-12.56231100	1.25119000	-2.07672100	C	16.26413500	-2.61608400	-2.08370400
C	-8.83306400	3.60368200	-3.57648300	H	14.76685800	-4.02086500	-2.64279400
H	-9.85411100	3.97286600	-3.69633300	C	12.84832400	-3.79742500	-4.25408600
H	-8.31033500	3.73848400	-4.52597400	O	17.86166100	-0.95979000	-1.54777200
C	-8.13396900	4.41784500	-2.49943600	C	10.31052400	0.65658300	4.10834100
C	-8.85271800	4.95332900	-1.42612500	H	13.04865000	4.16748500	2.06679700
H	-9.92216500	4.78838300	-1.40627800	H	12.65008300	5.65615900	1.16713200
C	-8.22140300	5.65772300	-0.39468100	H	11.60312600	5.11819700	2.50689500
C	-6.82643200	5.83721600	-0.41300900	C	7.90791300	-1.16338600	1.15851300
C	-6.10507700	5.31366600	-1.49446600	C	16.97696000	-3.58979800	-1.15847500
H	-5.03297500	5.47316900	-1.51222700	H	12.56376400	-4.10627400	-3.23989400
C	-6.74178700	4.61948100	-2.53425800	H	11.97418400	-3.85528900	-4.90516800
C	-6.10971900	6.54333100	0.72590900	H	13.62194400	-4.47954100	-4.62873200
H	-5.23619300	7.06273300	0.32535400	C	18.42186600	0.32459800	-1.76265000
H	-6.77534600	7.29177300	1.16067200	H	9.37216400	0.16986100	4.38587100
C	-4.78734300	4.60781400	-3.93767200	H	10.49336700	1.46241300	4.82359100
H	-4.83395500	5.69954300	-4.01201700	C	11.43940400	-0.35836700	4.19884700
H	-4.01353800	4.32530000	-3.21392000	H	8.50374100	-1.51222200	0.30526600
C	-10.30975800	6.17725000	0.66481000	H	7.46974000	-2.02468800	1.66641300
H	-10.70226600	5.15375800	0.71055100	H	7.09942500	-0.52098800	0.78340400
H	-10.62125500	6.72196500	1.55669600	C	16.42140000	-3.54459400	0.25596800
O	-3.58588800	5.17644900	0.66001800	H	18.04202600	-3.34802600	-1.13233800
O	-6.94199400	3.97423600	4.90890300	H	16.87248200	-4.60230900	-1.55516900
O	-2.91021800	0.91540500	3.18934100	H	17.74818400	1.12406600	-1.42774200
O	-7.86129300	-1.11316000	4.65973600	H	19.33910900	0.35755500	-1.17217900
O	-4.94174000	-3.13607500	0.58128000	H	18.66800400	0.48707800	-2.81958800
O	-10.35719300	-1.97592700	0.25649200	C	11.19987600	-1.71334800	3.95568900
O	-6.96125300	-1.01223100	-3.83397000	C	12.75356300	0.04130300	4.49686700
O	-10.87374800	2.48287600	-2.03324400	C	15.35882400	-4.37972300	0.64730700
O	-6.06082900	4.06026600	-3.59458300	C	16.93137100	-2.64244600	1.19243300
O	-8.88738800	6.19471300	0.67344000	H	10.18624600	-2.00966300	3.71731900
H	-10.71132300	6.67839200	-0.22377600	C	12.23245600	-2.65472300	3.98422000
H	-4.53117300	4.18685800	-4.91080200	C	13.78581100	-0.90056000	4.52712700
C	-12.57506000	-1.79749400	1.06586500	O	12.93995600	1.38183000	4.74954300
C	-13.58592500	-2.72755400	1.00857100	C	14.82933700	-4.28909700	1.93788300
N	-13.09237500	-0.58459100	0.69411500	O	14.89682000	-5.26083600	-0.30233200

H	-13.61139400	-3.77850100	1.24511800	C	16.39816600	-2.54772000	2.48130400
N	-14.35190100	-0.71348800	0.41348000	H	17.74190600	-1.99696000	0.87848100
N	-14.68155900	-2.02578300	0.60206000	C	13.54963500	-2.25263900	4.26485700
C	-16.01032500	-2.47051400	0.38804900	O	12.04253100	-3.99812800	3.74776600
C	-17.05811400	-1.53353100	0.41559200	H	14.80534400	-0.59977100	4.73297200
C	-16.27027200	-3.82596200	0.15436100	C	14.23226500	1.81406800	5.14015500
C	-18.36074100	-1.95529400	0.21686600	C	15.32681500	-3.37379900	2.86825700
H	-16.82823500	-0.49079900	0.59508300	H	13.99808900	-4.91270900	2.24156900
C	-17.58088900	-4.24429700	-0.03352300	C	13.89834200	-6.19257800	0.07745900
H	-15.46050300	-4.54441900	0.09982600	O	16.86470900	-1.67208500	3.43310700
C	-18.63831600	-3.32046700	-0.00424000	C	14.69711800	-3.25044700	4.24657800
H	-19.18101600	-1.24821800	0.23897300	C	10.71802600	-4.46326900	3.56072600
H	-17.81011900	-5.28847900	-0.21602000	H	14.56977200	1.30863300	6.05388500
N	-19.91717400	-3.85890300	-0.20424500	H	14.97400200	1.64803000	4.34823300
N	-20.86997100	-3.01826700	-0.17679600	H	14.14809700	2.88487100	5.33439000
C	-22.14099600	-3.59465100	-0.37468800	H	12.96241200	-5.69358000	0.36044900
C	-22.37758500	-4.97006200	-0.57810800	H	13.72078200	-6.81954400	-0.79806200
C	-23.22322100	-2.69775800	-0.36182900	H	14.23035400	-6.82384600	0.91134900
C	-23.67206500	-5.43541900	-0.76227700	C	18.00612600	-0.89005600	3.12238400
H	-21.53338300	-5.64832500	-0.58947800	H	15.45791900	-2.93378600	4.96379800
C	-24.52490400	-3.15049700	-0.54535300	H	14.32816600	-4.23002300	4.55975100
H	-23.01820900	-1.64439100	-0.20769100	H	10.25826500	-4.03503800	2.66020300
C	-24.71911200	-4.51476300	-0.74459700	H	10.79024400	-5.54632300	3.44549900
H	-23.85440600	-6.49621900	-0.91788000	H	10.08200000	-4.23524400	4.42582300
H	-25.35652500	-2.45105800	-0.53325000	H	18.23228700	-0.31432500	4.02150000
N	-26.11622200	-5.01960700	-0.91419900	H	18.86982700	-1.51790100	2.87000900
H	-26.59400700	-5.16178000	-0.01417000	H	17.81254500	-0.20061600	2.29054500
H	-26.68615800	-4.36525700	-1.46274700	H	-6.64740600	1.61646500	-1.83648300
				H	-6.30888100	1.52169800	-0.20289100
				H	-6.07008000	2.94926000	-1.03657200

Table S4: Optimized Cartesian structures of monomer **1** at the B3LYP/6-31G(d,p) level.

**Monomer 1 (neutral)**							
C	1.95384100	6.41897300	0.85586600	H	-4.35741300	6.71503700	1.82130900
C	0.63127800	6.80362400	1.12943200	O	2.49809600	6.89404300	-0.31879700
C	0.04577800	6.35637800	2.31734800	O	0.19615300	5.06341200	4.38798500
H	-0.97926400	6.64656500	2.51060700	O	4.50931700	2.84412400	2.07907200
C	0.73789500	5.53243000	3.20884700	O	0.27169800	-0.08249400	4.14773700
C	2.05516700	5.13595100	2.92814400	O	2.85890800	-1.38881300	-0.35676600
C	2.64741800	5.59872900	1.74953000	O	-2.65502100	-1.58746400	0.24114400
H	3.65768300	5.27318700	1.53587200	O	-0.15959200	0.08749500	-4.20279700
C	3.84074700	6.55421500	-0.61405200	O	-4.54881700	2.67250400	-1.99957700
H	4.07547000	7.04115800	-1.56271800	O	-0.37946200	5.23840400	-4.20909700
H	4.53222400	6.91945300	0.15672800	O	-2.77677900	6.71837200	0.57283000
H	3.97358800	5.46998800	-0.72218700	H	-4.80986200	6.64436500	0.09788300
C	-1.11196300	5.48121800	4.73049100	H	1.14794000	5.34751300	-5.51708200
H	-1.85477800	5.14345800	3.99586700	C	-4.70599100	-1.64004000	1.42156100
H	-1.33275100	5.02633500	5.69826700	C	-5.54318800	-2.72638500	1.30105900
H	-1.17979100	6.57324800	4.82389900	N	-5.47192700	-0.50773400	1.36951200
C	3.46666000	2.09231600	2.56810600	H	-5.35108600	-3.78591500	1.26046800
C	2.61137800	2.73197300	3.48123100	N	-6.72279600	-0.83229800	1.22695900
C	1.54550000	2.00223900	4.01496300	N	-6.78913000	-2.19333600	1.18109700
H	0.88562000	2.51166200	4.70607300	C	-8.03975000	-2.85266400	1.03417600
C	1.31276800	0.67450200	3.64765100	C	-9.09952000	-2.18101000	0.40711200
C	2.15836800	0.03824800	2.72509100	C	-8.20759900	-4.15585800	1.51023900
C	3.23465600	0.76251900	2.20356700	C	-10.32195900	-2.81652200	0.25547500
H	3.87280000	0.27017800	1.48060300	H	-8.94509000	-1.16991700	0.05051500
C	5.44200700	2.21354200	1.21175500	C	-9.43352100	-4.79081800	1.34190100
H	4.97966400	1.86931200	0.28026700	H	-7.39873900	-4.66295900	2.02498000
H	6.19537200	2.96887000	0.98053800	C	-10.50132000	-4.13352600	0.71682200
H	5.93088200	1.35970700	1.69812600	H	-11.15303800	-2.31625700	-0.22698200
C	-0.57160200	0.50910300	5.11861300	H	-9.59161400	-5.80185100	1.70194100
H	-1.10380800	1.38281300	4.72026200	N	-11.69994500	-4.87743600	0.60654300
H	-1.29873400	-0.25635400	5.39741500	N	-12.64750300	-4.25788700	0.03928800
H	-0.01200400	0.81444300	6.01269500	C	-13.84805500	-4.97317300	-0.08232500
C	2.80978200	4.19326300	3.85277600	C	-14.05784600	-6.29159000	0.37053400
H	3.87554000	4.43145700	3.81146200	C	-14.90657400	-4.29747100	-0.71076800
H	2.47070600	4.34951800	4.87987200	C	-15.28407100	-6.90401900	0.19626300
C	1.89288300	-1.38975100	2.27215300	H	-13.23873100	-6.81144100	0.85396000
H	2.84464500	-1.86773400	2.02767200	C	-16.13873400	-4.90659200	-0.88909200
H	1.43970600	-1.94902900	3.09473200	H	-14.73210900	-3.28352500	-1.05640600
C	-1.28016800	-1.52127100	0.12916400	C	-16.34980700	-6.22400300	-0.43783900
C	-0.41447100	-1.51834000	1.22625400	H	-15.44050600	-7.91978600	0.55108700
H	-0.79634500	-1.52841800	2.23933200	H	-16.94916500	-4.37001100	-1.37498300
C	0.97258700	-1.45806200	1.06282900	N	-17.58646800	-6.82712500	-0.56511300
C	1.48292300	-1.41989000	-0.24452700	H	-18.21788100	-6.44231100	-1.25186900
C	0.61728700	-1.41904100	-1.34150100	H	-17.61614200	-7.83391200	-0.50308000
H	0.99747100	-1.35276500	-2.35312800	C	4.90856000	-1.23489100	-1.52679500
C	-0.77053200	-1.45957600	-1.17755300	C	5.81183300	-2.26265000	-1.37138000
C	3.42283800	-1.27904600	-1.66152100	N	5.60252200	-0.05626300	-1.51606900
H	3.07981100	-0.36143800	-2.15468300	N	7.02186900	-1.64916400	-1.27181900
H	3.11895300	-2.13571600	-2.27875800	H	5.69276200	-3.33341000	-1.35129400
C	-3.21998700	-1.58658800	1.54969000	N	6.87146900	-0.29735400	-1.36397800
H	-2.93706300	-0.67448500	2.08947700	C	8.31070300	-2.22506400	-1.10503600
H	-2.85589000	-2.45102800	2.12161000	C	9.43611300	-1.53593300	-1.56607600

C	-1.69507000	-1.39177600	-2.38367500
H	-2.61670100	-1.93510600	-2.16168200
H	-1.21118500	-1.88630500	-3.22987700
C	-1.24010400	0.76077800	-3.66783700
C	-2.04392100	0.03711500	-2.77271500
C	-3.15760400	0.67474600	-2.21754300
H	-3.76325900	0.11552600	-1.51535500
C	-3.46684500	2.00374800	-2.52311200
C	-2.65356800	2.73095200	-3.40865100
C	-1.54983300	2.08786000	-3.97574100
H	-0.92301800	2.66417900	-4.64473900
C	0.64203100	0.76712300	-5.15112100
H	1.12690600	1.65176400	-4.71795000
H	1.40925800	0.05682500	-5.46606000
H	0.05963600	1.07820800	-6.02849500
C	-5.44429500	1.94924700	-1.16618500
H	-4.96361500	1.58398900	-0.25232900
H	-6.23818300	2.64954600	-0.89953800
H	-5.88484600	1.09442000	-1.69536500
C	-2.93693400	4.19327000	-3.71537600
H	-4.01467500	4.36718200	-3.66457600
H	-2.60918900	4.41422400	-4.73422700
C	-2.23697900	5.13636200	-2.74907700
C	-2.85323100	5.51035400	-1.55143100
H	-3.84202700	5.11632600	-1.35335400
C	-2.20750600	6.32917800	-0.62140600
C	-0.91033700	6.80277600	-0.87633900
C	-0.30163800	6.44412000	-2.08251700
H	0.70426500	6.80236000	-2.26169900
C	-0.94573100	5.62157500	-3.01061700
C	-0.16425800	7.64733600	0.14520100
H	0.51519200	8.32318800	-0.38025900
H	-0.88146500	8.25839300	0.69908300
C	0.90228300	5.74532800	-4.53033900
H	0.90740900	6.84253200	-4.57481100
H	1.66382200	5.41815200	-3.81025800
C	-4.09622200	6.28636100	0.85168100
H	-4.16419200	5.19236700	0.91042900
C	8.44545000	-3.47200800	-0.47807900
C	10.69341000	-2.10448600	-1.39983800
H	9.31149500	-0.57147700	-2.04245500
C	9.70276600	-4.03858500	-0.32916500
H	7.57148400	-3.98374000	-0.08857800
C	10.84332400	-3.35839500	-0.79058900
H	11.58489400	-1.59323700	-1.74745900
H	9.82838500	-5.00012800	0.15388400
N	12.17062200	-3.83753900	-0.68530700
N	12.27251200	-4.97820400	-0.14500500
C	13.57966500	-5.47325300	-0.02507900
C	13.70734900	-6.73937900	0.56859700
C	14.74425700	-4.80246900	-0.45074400
C	14.95046100	-7.32902700	0.73630600
H	12.80341600	-7.24618200	0.89124600
C	15.98634700	-5.38462600	-0.28565900
H	14.64289400	-3.82565200	-0.90947300
C	16.11494000	-6.66046000	0.31103900
H	15.03361500	-8.30821600	1.20009600
H	16.88092200	-4.86016600	-0.61244200
N	17.36437600	-7.21359200	0.51553800
H	18.12384600	-6.85616000	-0.04458100
H	17.40752600	-8.20923400	0.67408000

Table S5: Optimized Cartesian structures of dimer **1** at the B3LYP/6-31G (d,p) level.

**Dimer 1 (neutral)**							
C	-8.42929900	5.09141300	2.54652500	H	8.48848800	0.62745800	-3.91494200
C	-9.50586400	5.94868000	2.29519100	O	8.45704300	0.44293600	-1.85066400
C	-10.54297600	5.98710900	3.23740600	C	9.79569000	0.71370000	-1.62488300
H	-11.38663100	6.63365400	3.03390300	C	10.80361900	0.41099300	-2.54438600
C	-10.51152100	5.19556000	4.38668300	C	10.11115800	1.31259400	-0.39637500
C	-9.42623400	4.33196800	4.63603000	H	10.57705300	-0.09447600	-3.47459000
C	-8.39686600	4.29884000	3.69596100	C	12.14459700	0.70183600	-2.28118400
H	-7.56288100	3.62080100	3.85366600	C	11.45148200	1.61984800	-0.13964700
C	-6.18157700	5.64839100	2.03222900	C	9.04601200	1.58431100	0.65457600
H	-5.45162300	5.49617200	1.23416600	C	13.23229700	0.30702900	-3.26841600
H	-6.34189900	6.72357900	2.18130100	C	12.45786400	1.32889200	-1.06370500
H	-5.79512800	5.21801000	2.96378100	H	11.68488400	2.06805200	0.81803000
C	-12.59482100	6.09403400	5.16257300	H	8.08520400	1.74615500	0.15982900
H	-13.16372100	5.88170200	4.24804400	H	9.30146100	2.50080700	1.19215400
H	-13.24260600	5.95141900	6.02928800	C	8.90520800	0.44419000	1.65171400
H	-12.25464500	7.13721600	5.13463600	C	13.78974800	-1.08291000	-2.99997500
C	-8.95775800	0.98227700	5.30360000	H	14.04726100	1.03307000	-3.21518900
C	-9.87511800	2.01887300	5.53584200	H	12.82069500	0.34028700	-4.28053700
C	-11.24084600	1.73822400	5.42859900	O	13.78916600	1.62823200	-0.86312100
H	-11.93727200	2.54589000	5.61916700	C	9.64999000	0.42477900	2.84110100
C	-11.69706200	0.46839100	5.07198600	C	8.05372800	-0.63286000	1.38556000
C	-10.78135700	-0.56072900	4.78809400	C	13.18625700	-2.22330600	-3.55357100
C	-9.41760000	-0.28885600	4.93351800	C	14.89922500	-1.25788900	-2.16730900
H	-8.71067700	-1.08626800	4.73346600	C	14.16381100	2.22287500	0.37806800
C	-6.69381400	0.23355300	5.59532600	C	9.54065500	-0.65499500	3.72178500
H	-6.58404700	-0.36268800	4.68420200	O	10.47107600	1.51078700	3.06721300
H	-5.73991300	0.70752500	5.83556300	H	7.49380300	-0.61585200	0.45839600
H	-6.98593900	-0.42779100	6.42118200	C	7.94733800	-1.71394900	2.26477100
C	-13.98551300	1.13683100	5.30083900	C	13.68291100	-3.49517900	-3.25739400
H	-13.92302900	1.99487800	4.61878900	O	12.10499300	-2.00166200	-4.38298900
H	-14.96552200	0.66706300	5.19505700	C	15.39661400	-2.53036200	-1.86970600
H	-13.87007600	1.49545100	6.33220300	H	15.35003200	-0.37508700	-1.73182300
C	-9.40229400	3.42699400	5.85783800	H	13.90995400	1.56022400	1.21432200
H	-8.38372200	3.37767800	6.24799700	H	13.63147800	3.17365200	0.51755300
H	-10.03980200	3.85744800	6.63345900	C	8.70270200	-1.73921600	3.44930200
C	-11.25751700	-1.92690400	4.31590200	H	10.13000400	-0.69304400	4.62909200
H	-10.45914800	-2.65287900	4.48620100	C	11.19034000	1.55808000	4.28644600
H	-12.12188200	-2.23403700	4.91282100	O	7.12267000	-2.79473300	2.04727100
C	-13.34334000	-1.70516700	1.10318000	C	14.78147000	-3.67170700	-2.41154200
C	-12.97593400	-1.73431900	2.44928300	H	13.20981900	-4.38353400	-3.65698700
H	-13.70258400	-1.54118600	3.22708900	C	11.50630900	-3.12272500	-5.00689500
C	-11.64758000	-1.95799900	2.84345700	O	16.48303800	-2.75114100	-1.05619300
C	-10.70393400	-2.20171300	1.83922600	C	8.64434000	-2.93029500	4.39346100
C	-11.08505000	-2.21312400	0.49461000	H	11.90241700	0.72712100	4.37304300
H	-10.34217700	-2.41671200	-0.26886900	H	11.73999300	2.50136800	4.27839300
C	-12.39054100	-1.94228200	0.09438900	H	10.51942200	1.54088300	5.15525100
C	-8.42742100	-1.53633800	1.68398400	C	6.34022600	-2.81262600	0.86742800
H	-8.49658500	-0.60594900	2.26013400	C	15.26879200	-5.06842400	-2.05904200
H	-8.63686200	-1.29973400	0.63524700	H	11.07995800	-3.81960500	-4.27345400
C	-15.61150100	-1.10754800	1.63863600	H	10.70454500	-2.72934100	-5.63517400
H	-15.28893300	-0.22545200	2.20512800	H	12.22126000	-3.66781800	-5.63719000
H	-15.76123600	-1.93462200	2.34586700	C	17.18229900	-1.62229000	-0.54910400

C	-12.75088800	-1.85317400	-1.38040600	H	7.63477800	-3.34887200	4.38228900
H	-13.80242100	-2.11567400	-1.51651400	H	8.85263200	-2.58705400	5.41005000
H	-12.14986900	-2.57675900	-1.93720700	C	9.63611000	-4.02122100	4.01982900
C	-11.25510000	-0.09652300	-2.46568800	H	6.96558100	-2.80753500	-0.03484100
C	-12.50903500	-0.46101500	-1.94575700	H	5.76069400	-3.73656100	0.89567200
C	-13.52176400	0.50206500	-1.92087400	H	5.64962500	-1.95959500	0.82488100
H	-14.47870700	0.21980900	-1.49942500	C	14.58490400	-5.62794200	-0.82112400
C	-13.30701700	1.80345000	-2.38758400	H	16.34752900	-5.03796200	-1.88646900
C	-12.05216100	2.16517800	-2.90296700	H	15.08306500	-5.73524200	-2.90462700
C	-11.04119800	1.20068100	-2.93642700	H	16.55830400	-1.00423400	0.10556200
H	-10.07369800	1.49839600	-3.32239400	H	18.02012200	-2.02142000	0.02559700
C	-9.02160000	-0.75672200	-3.03033600	H	17.57346200	-0.99396500	-1.35959400
H	-8.51934000	0.04075600	-2.46716000	C	9.26303500	-5.05870200	3.16063500
H	-8.42465500	-1.66892500	-2.97065900	C	10.95684000	-3.99644700	4.49619500
H	-9.10271400	-0.45227900	-4.08209600	C	13.39924600	-6.37391000	-0.91793200
C	-15.58856600	2.42788600	-1.97949100	C	15.10442200	-5.37488000	0.45160500
H	-15.63720200	2.09442600	-0.93742500	H	8.24592300	-5.06181400	2.78992800
H	-16.19205800	3.32966900	-2.09804100	C	10.17402000	-6.03979700	2.75979200
H	-15.99739100	1.63718800	-2.62090300	C	11.86677600	-4.97936900	4.09849100
C	-11.77367900	3.57934200	-3.38675300	O	11.27673800	-2.96647600	5.35586400
H	-12.70145500	4.02171000	-3.75786400	C	12.76159300	-6.83462800	0.23702100
H	-11.06797000	3.53304500	-4.21992000	O	12.92689000	-6.60850400	-2.19269800
C	-11.20140000	4.46688300	-2.29343800	C	14.46347700	-5.83002700	1.60681300
C	-12.05433900	5.10932600	-1.38296500	H	16.00933800	-4.78383200	0.51407300
H	-13.12025100	4.96269500	-1.50412600	C	11.49924800	-6.00500700	3.22256600
C	-11.55153000	5.87763600	-0.33162600	O	9.85046500	-7.07528000	1.90850400
C	-10.16053600	6.02734400	-0.15219100	H	12.89468100	-4.95528000	4.43807500
C	-9.32160600	5.40787500	-1.07329600	C	12.58571500	-2.93313000	5.89428900
H	-8.24725900	5.49544300	-0.94960300	C	13.27194200	-6.56657200	1.51062800
C	-9.82563200	4.63620500	-2.12239100	H	11.83390100	-7.38982000	0.17581800
C	-9.58756400	6.79076100	1.03015900	C	11.76343200	-7.40214500	-2.33454900
H	-8.58818600	7.14390100	0.76177800	O	14.93688300	-5.59575300	2.88089600
H	-10.20539600	7.67028700	1.23119500	C	12.52545600	-7.02493000	2.75400900
C	-8.32059000	4.81695700	-3.94930200	C	8.50712900	-7.18493800	1.47385200
H	-9.07590500	5.22816600	-4.63019900	H	12.81153600	-3.83890800	6.47234200
H	-7.77324300	5.64577200	-3.48505600	H	13.34649900	-2.81483100	5.11170900
C	-13.75427200	6.40890300	0.44411800	H	12.61863200	-2.06829600	6.56002300
H	-14.08455200	5.36715500	0.53778600	H	10.89031300	-6.93682200	-1.85849700
H	-14.18580500	6.99925000	1.25436700	H	11.58290400	-7.48777100	-3.40797700
O	-7.38839100	5.00197900	1.61829600	H	11.90135300	-8.40658300	-1.91290600
O	-11.51283500	5.19461300	5.32961200	C	16.16032400	-4.89532200	3.01685200
O	-7.62838300	1.30060400	5.44239900	H	13.24317800	-7.20419100	3.55831400
O	-13.03281800	0.13966800	4.98201700	H	12.02043600	-7.96988200	2.53920900
O	-9.38951500	-2.48080000	2.18491700	H	8.19742300	-6.31579400	0.87888900
O	-14.62913100	-1.45889900	0.66703300	H	8.46180700	-8.07980000	0.85014900
O	-10.28613500	-1.07739200	-2.47769100	H	7.81436000	-7.30017100	2.31769700
O	-14.26907300	2.78658500	-2.37442700	H	16.36199300	-4.84220000	4.08857900
O	-8.92586200	3.97383600	-2.96472500	H	16.98745200	-5.42120300	2.52208300
O	-12.34655200	6.52684100	0.57934200	H	16.09601800	-3.87753500	2.61075200
H	-14.10325400	6.80841600	-0.51645200	H	-7.57143800	3.13939500	-1.52858300
H	-7.62193800	4.19517500	-4.51325000	H	-7.07791300	3.43180300	0.08978200
C	-16.88515300	-0.79726800	0.92571000	C	-7.05293500	-2.10280300	1.79863300
C	-17.91405900	-1.63198300	0.55111900	C	-6.26210800	-2.67663300	0.82928800
N	-17.19129800	0.46738900	0.50362100	N	-6.33335700	-2.06953900	2.96093900
H	-18.08563800	-2.68422100	0.70795600	N	-5.08967200	-2.96003900	1.45772800

N	-18.34029900	0.45846100	-0.10593000	H	-6.41806300	-2.85688400	-0.22158200
N	-18.80210800	-0.82353900	-0.08784700	N	-5.15281100	-2.58018000	2.76266500
C	-20.05329600	-1.15254100	-0.67781600	C	-3.87935000	-3.44322400	0.88833300
C	-21.04829700	-0.16856600	-0.77089400	C	-2.65537800	-2.97452200	1.38518600
C	-20.28325000	-2.44419100	-1.15911200	C	-3.91768800	-4.32651300	-0.19325300
C	-22.27217900	-0.48152500	-1.34169300	C	-1.47522100	-3.34960800	0.76364500
H	-20.84306800	0.82609500	-0.39394500	H	-2.65411400	-2.30065200	2.23360400
C	-21.51891200	-2.75311100	-1.71776300	C	-2.72849300	-4.69173300	-0.81667200
H	-19.50084900	-3.19434400	-1.11931800	H	-4.86533800	-4.72096000	-0.54494800
C	-22.52380700	-1.78170800	-1.81678500	C	-1.50105700	-4.18763800	-0.36649800
H	-23.05544800	0.26260800	-1.42236300	H	-0.52041200	-2.97019600	1.10661100
H	-21.72493500	-3.74731100	-2.09967700	H	-2.72910400	-5.35371800	-1.67618800
N	-23.73612300	-2.21114900	-2.40684100	N	-0.36942700	-4.52138000	-1.14960300
N	-24.62463200	-1.31216600	-2.48532200	N	0.69083100	-3.93005200	-0.78459400
C	-25.83670100	-1.71182200	-3.06668000	C	1.81288700	-4.12220500	-1.59596300
C	-26.11677800	-3.00502600	-3.55344600	C	1.86697300	-4.93844500	-2.74458200
C	-26.83051200	-0.72398200	-3.16026300	C	2.94234900	-3.35631900	-1.25760400
C	-27.34760400	-3.29042000	-4.11220800	C	2.99441900	-4.94410700	-3.54412900
H	-25.34747700	-3.76522000	-3.48165400	H	0.99752000	-5.53275200	-3.00213300
C	-28.06697400	-1.00381300	-3.72025200	C	4.07159000	-3.35587300	-2.05645800
H	-26.60216800	0.26803200	-2.78373200	H	2.88666600	-2.73606400	-0.36848200
C	-28.34816600	-2.29515900	-4.20767400	C	4.10745700	-4.12396000	-3.23963700
H	-27.55848400	-4.29061700	-4.48263800	H	3.02110400	-5.55799800	-4.44142500
H	-28.82691800	-0.22970100	-3.78357600	H	4.92563300	-2.73640000	-1.80398000
N	-29.59158100	-2.60200400	-4.72477200	N	5.21665100	-4.08927700	-4.06035500
H	-30.16689100	-1.83198000	-5.03192600	H	5.72425400	-3.20791400	-4.06885900
H	-29.65885000	-3.42141700	-5.30981300	H	5.07452300	-4.45759400	-4.99082400
N	-6.88581300	2.98238800	-0.79846900	C	15.63859200	2.44997100	0.36403700
C	-5.59761900	2.70579600	-1.15143300	C	16.36617600	3.49401200	-0.16232800
C	-4.55332600	2.74028100	-0.18827200	N	16.51713700	1.55178300	0.90451200
C	-5.26773900	2.34288000	-2.47944800	N	17.66416500	3.17249400	0.08646800
C	-3.25201700	2.44647100	-0.53930900	H	16.07081400	4.40978500	-0.64740200
H	-4.79867000	2.99160800	0.84000000	N	17.73458400	1.97914400	0.74149700
C	-3.96145400	2.04207100	-2.82069800	C	18.84621600	3.89517400	-0.23268500
H	-6.05581600	2.29925900	-3.22620700	C	19.99305800	3.71698000	0.55477900
C	-2.92663000	2.09312200	-1.86811400	C	18.86031700	4.77135700	-1.32129800
H	-2.45716200	2.47421500	0.19749000	C	21.14917100	4.41974600	0.25300500
H	-3.70233700	1.76225400	-3.83732700	H	19.95716500	3.02792000	1.38989800
N	-1.64951700	1.76633700	-2.32725200	C	20.02053600	5.48256300	-1.60852900
N	-0.71423100	1.86402900	-1.47366700	H	17.98446500	4.88106500	-1.95160900
C	0.55707500	1.48225000	-1.96031700	C	21.17387800	5.31712300	-0.83032300
C	0.80825300	0.93525800	-3.23282500	H	22.04598900	4.29797700	0.84851400
C	1.62547900	1.64661600	-1.06725600	H	20.06025400	6.16861600	-2.44788700
C	2.09349800	0.56296800	-3.59727500	N	22.29345200	6.08949700	-1.22068900
H	-0.02273300	0.80890100	-3.91633400	N	23.32064700	5.91953800	-0.49985000
C	2.91601300	1.26793000	-1.42092400	C	24.44577200	6.67241700	-0.86659100
H	1.41518800	2.05713700	-0.08541500	C	24.50242200	7.57426700	-1.94872600
C	3.14726400	0.72562000	-2.68789400	C	25.59142200	6.49386400	-0.07429600
H	2.29648900	0.13316400	-4.57096700	C	25.66577100	8.26825500	-2.22041400
H	3.72633000	1.36327000	-0.70597600	H	23.61649800	7.71106600	-2.55807900
N	4.45954800	0.30356200	-3.05158700	C	26.76122200	7.18750500	-0.34129800
C	5.66211200	0.74950500	-2.59125700	H	25.53484400	5.79777300	0.75652100
N	4.63536500	-0.72847800	-3.90856400	C	26.81932900	8.08935300	-1.42173000
C	6.59015900	-0.06417900	-3.20160700	H	25.70411200	8.95940500	-3.05884400
H	5.76559300	1.58123600	-1.91562500	H	27.64058700	7.03669400	0.27899700

N	5.92144600	-0.95162300	-3.99475200	N	27.99189900	8.75146400	-1.72965300
C	8.08359300	-0.03377000	-3.13426900	H	28.68757600	8.82093400	-1.00201100
H	8.47737000	-1.04169900	-3.32288200	H	27.91820800	9.57721100	-2.30491700
